# The importance of batch sensitization in missing value imputation

**DOI:** 10.1038/s41598-023-30084-2

**Published:** 2023-02-21

**Authors:** Harvard Wai Hann Hui, Weijia Kong, Hui Peng, Wilson Wen Bin Goh

**Affiliations:** 1 Lee Kong Chian School of Medicine, Nanyang Technological University, Singapore, 636921 Singapore; 2 School of Biological Sciences, Nanyang Technological University, Singapore, 637551 Singapore; 3 Center for Biomedical Informatics, Nanyang Technological University, Singapore, 636921 Singapore

**Keywords:** Data integration, Data processing, Statistical methods

## Abstract

Data analysis is complex due to a myriad of technical problems. Amongst these, missing values and batch effects are endemic. Although many methods have been developed for missing value imputation (MVI) and batch correction respectively, no study has directly considered the confounding impact of MVI on downstream batch correction. This is surprising as missing values are imputed during early pre-processing while batch effects are mitigated during late pre-processing, prior to functional analysis. Unless actively managed, MVI approaches generally ignore the batch covariate, with unknown consequences. We examine this problem by modelling three simple imputation strategies: global (M1), self-batch (M2) and cross-batch (M3) first via simulations, and then corroborated on real proteomics and genomics data. We report that explicit consideration of batch covariates (M2) is important for good outcomes, resulting in enhanced batch correction and lower statistical errors. However, M1 and M3 are error-generating: global and cross-batch averaging may result in batch-effect dilution, with concomitant and irreversible increase in intra-sample noise. This noise is unremovable via batch correction algorithms and produces false positives and negatives. Hence, careless imputation in the presence of non-negligible covariates such as batch effects should be avoided.

## Introduction

Missing values (MV) and batch effects (BE) are both endemic problems in high-dimensional biological data analyses. MVs relate to particular information points being present in some samples but not in others^1^. In biology, MVs arise from several factors, with some examples being poor instrument resolution, or the biological moiety having an abundance below instrument detection limit^2^. We may categorize MVs into three groups, according to the mechanism by which they occur. Missing completely at random (MCAR) refers to MVs which result from no specific variables nor biases and are thus independent of abundance. MVs that are missing at random (MAR) are those which may be dependent on other observed data, but not a result of the MVs itself. Missing not at random (MNAR), on the other hand, refers to MVs which are due to abundances below the limit of detection, and thus depend on the MVs themselves^3^. For simplicity, this study will only tackle MVs that are MCAR in nature.

The presence of MVs may impede performance of downstream analyses, as the observed values without the MVs may not be a true representation of the actual dataset^4^. One way to handle MVs is to conduct missing value imputation (MVI), which replaces MVs with either a constant or an estimated value based on other observations. One thing to note is that MVI is usually carried out only when necessary, that is, when downstream analyses demand a complete dataset and omitting MVs is not an option (e.g., when the dataset is too small). This is because most high-dimensional biological data contain a mixture of MV types, and there is no fool proof method to precisely determine the exact type and proportion of MVs present in the dataset^5^. Even then, many MVI methods exist, and their performances can vary greatly. In any case, perceived advantage of MVI when done correctly, is that it can improve statistical power and serve to provide a more accurate representation of the underlying data.

BE relates to technical sources of biases that may confound the true signal-of-interest, and can hinder proper analysis^6^. For example, the same protein sample may produce varying levels of abundance when subject to different machines. Batch-effect correction algorithms (BECAs) are algorithms which detect batch artifacts within datasets and attempts to reduce their effect^7^. To date, a large number of MVI methods and BECAs have been developed. Although neither are considered fully “solved” problems, many would concur that they are at least “manageable”, provided appropriate conditions are fulfilled. However, MVs and BEs are not mutually exclusive problems. Although typically treated separately, there are in fact, temporal dependencies between the two: In general, MVs are imputed during early phases of pre-processing (producing a pseudo-complete data matrix) while batch effects are dealt with during late pre-processing (on the pseudo-complete matrix), prior to functional analysis. This temporal dependency means that the efficacy of the batch correction is dependent on how MVI was performed. To be fair, most MVI methods were created not only for the sake of batch-effect prone high-dimensional biological data, but for many other domains as well. Batch covariates, which are not as common in those areas, are thus not considered in MVI methods. However, this does not mean that there is no functional way in which we can avoid or at the very least reduce the effect of batch factors when conducting MVI. Suppose we know early on that a batch (or some other important and non-negligible) co-variate exists, we hypothesize it would make more sense to impute using this information early on.

Surprisingly, a search in current literature using “Missing Value Imputation” and “Batch Effect” did not reveal any studies (or mention) examining confounding effects between the two. A few review articles did emerge, but discussed the topics as entirely separate entities (as a broad checklist of considerations for data processing). Hence, this work is the first to explore and evaluate how batch sensitization in MVI impacts batch correction (which subsequently impairs the ability to identify correct gene targets). Where both batch effects and missing values are present in data, we recommend caution, and to ensure that the batch factor is taken into consideration early in the data processing stage.

## Materials and methods

All methods were performed in accordance with the relevant guidelines and regulations.

### Batch effect correction algorithms (BECAs)

#### ComBat

ComBat is a widely used batch effect correction algorithm. It involves using an Empirical Bayes (EB) method to estimate the Location (mean) and Scale (variance) model parameters. These EB estimates are then used to adjust the data for batch effects. ComBat is known to be robust to outliers and perform well for small sample sizes^8^.

#### Surrogate variable analysis (SVA)

SVA is a batch correction method that is based on matrix factorisation. It assumes batch effects are induced by unmodeled factors. SVA borrows information across samples to estimate the large-scale effects of all unmodeled factors from the data. Sources of variation induced by unmodeled factors can then be removed, thereby removing batch effects^9^.

#### Harman

Harman is a batch correction method that is based on Principal Component Analysis (PCA) and constrained optimisation. It removes batch effects from datasets, with the constraint that the probability of overcorrection (i.e., removing genuine biological signal along with batch noise) is kept to a certain limit. In our case, the confidence limit is set to 0.95, which means that the probability of removing biological signals along with batch noise is 0.05^10^.

#### Batch mean centering (BMC)

BMC corrects batch effect by subtracting the batch mean from the data. This is equivalent to zero-centering each batch. Thus, after BMC adjustment, the mean of all samples in each batch is zero^11^.

### Batch effect detection methods

#### Principal component analysis (PCA)

PCA is often used as a dimensionality reduction technique for data compression and visualisation (on 2D/3D scatter plots). It reduces high dimensional data into lower numbers of linearly uncorrelated variables known as principal components (PCs). The ordering of the PCs are such that the first PC has the highest variance, and the second PC has the next highest variance, and so on. Combined with scatterplots, PCA can be used for visual inspection for batch effects. This is, however, not effective if the batch-correlated PC is not amongst the top PCs.

#### Guided PCA (gPCA)

A more informative version of PCA for detecting batch effects is gPCA, which is guided by a batch indicator matrix to look for batch effects in the data^12^. Typically, gPCA is performed on Y^T^X, where Y is a batch indicator matrix and X is the data matrix. gPCA provided 2 metrics, a delta, which is the proportion of total variance of the data that is induced by batch effects, and its associated p-value. Both delta and its associated p-value range from 0 to 1. If the delta is nearer to 1, the batch effect is large. A low *p*-value (< 0.05) supports the confidence of the estimated delta value^11, 12^. We found that the *p*-value is stable when the delta is high anyway, so we only use gPCA delta in this study^7^.

#### Imputation accuracy

Imputation accuracy measures similarity between imputed matrix and original matrix, and is determined via the root mean square error (RMSE):

$$RMSE=\sqrt[2]{\frac{1}{{n}_{miss}}\sum_{i=1}^{{n}_{miss}}{({y}_{i}^{miss}-{\dot{y}}_{i})}^{2}}$$

where ${y}_{i}^{miss}$ represents the true (but removed) data value i, and ${\dot{y}}_{i}$ is the imputed value. Since there are n_miss_ missing values, the RMSE is the square root of the average of the sum of deviations between the true and imputed values. The lower the RMSE, the better the imputation accuracy.

#### Imputation strategies (M1, M2 and M3)

**Figure 1:**
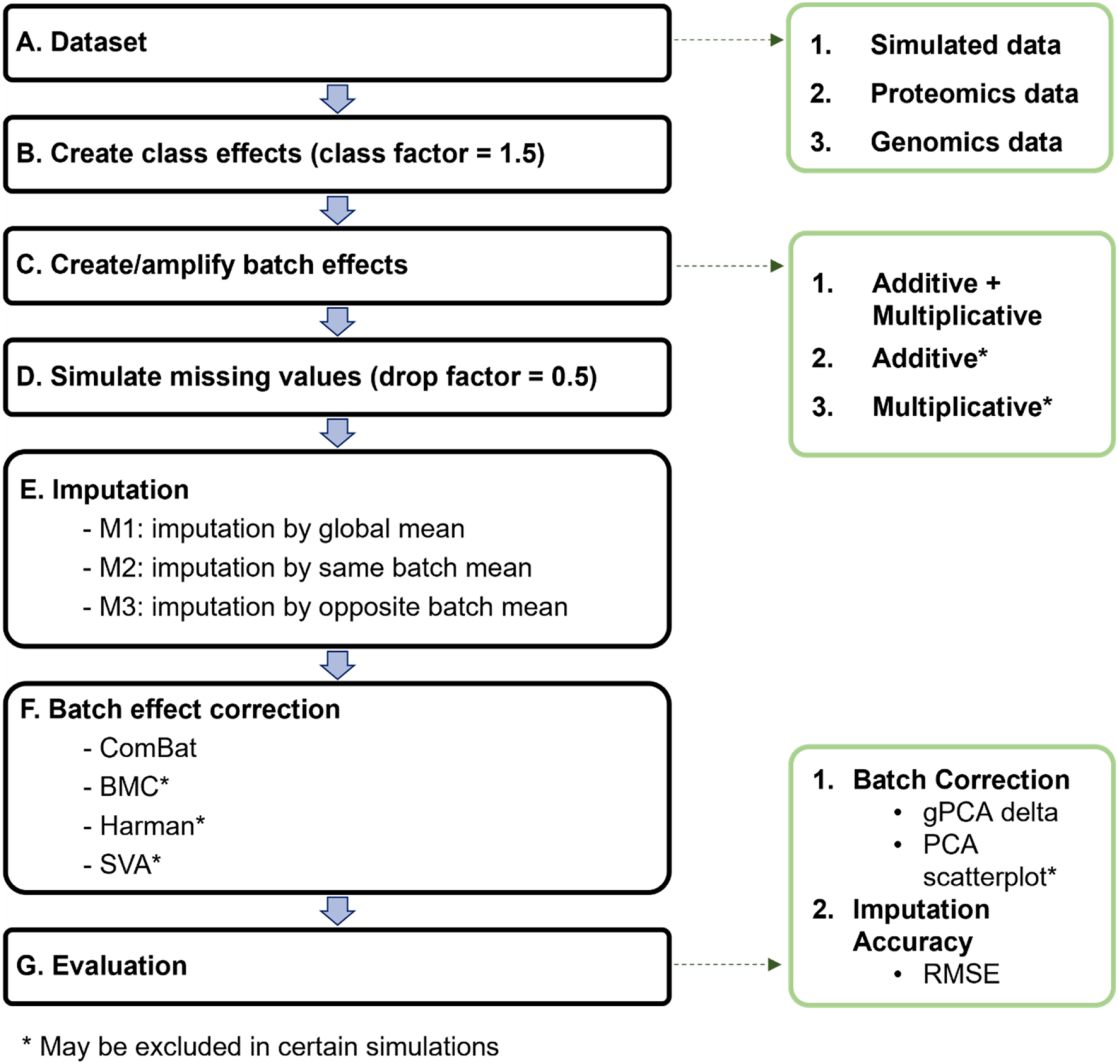
(**A**) 3 types of datasets are used for this analytical pipeline: simulated data for **1.** Initial Simulation, Renal Control dataset (RCC) for **2.** Proteomics Simulation and lastly, GDS4056/4057 combined dataset for **3.** Genomics Simulation. Analytical pipeline consists of (**B**) simulating class and (**C**) batch effects, followed by (**D**) introduction of missing values, (**E**) imputation, (**F**) batch correction and **G.** evaluation. (see Supplementary Methods for more detailed captions for each simulation).

To evaluate imputation strategies, we propose the following: M1 represents any global imputation strategy that does not account for the batch co-variate. M2 is our proposed batch-sensitized approach. M3 is a simulated worst-case scenario where only values from the opposite batch are used for imputation. To effect M1 to M3, we impute missing values with the global mean (M1) (i.e., mean of remaining values), same batch mean (M2) (i.e., mean of remaining values from the same batch) and opposite batch mean (M3) (i.e., mean of remaining values from opposite batch) (Fig. 1E).

#### Sophisticated MVI methods

To assess the applicability of our evaluations to other MVI methods, we briefly explored the effects of the aforementioned imputation strategies on two sophisticated MVI methods: k-nearest neighbour (KNN)^13^ and multivariate imputation by chained equations (MICE)^14^. We chose these two methods due to their widespread use, as well as their simplicity. These are not the immediate focus of our study but could serve as a gate for future works that aim to understand how presence of batch effects influence these complex imputation techniques, which in turn, affect BE correction. Information regarding the two methods can be found in the Supplementary Materials.

### Batch simulation strategies

#### Feature selection analysis

For initial analysis, we use simulated data for simplicity. A 20 × 20 matrix of normally distributed random numbers with a mean of 5 and a standard deviation (SD) of 1 was generated (Fig. 1A).

Due to the specific requirement for a class factor in Harman and SVA, class effects are simulated (Fig. 1B). 20 samples are split evenly into 2 classes (class 0 and 1), such that all odd samples (i.e., sample 1, 3, 5, 7, 9, 11, 13, 15, 17, 19) are in class 0 and even samples (i.e., sample 2, 4, 6, 8, 10, 12, 14, 16, 18, 20) are in class 1. Class effects are then loaded onto class 0 only by multiplying class 0 by a factor of 1.5.

After adding class effects, batch effects are simulated (Fig. 1C). 20 samples are split evenly into 2 technical batches, such that the first 10 samples are designated batch 0 while the last 10 samples are designated batch 1. This class and batch allocation ensures uniform distribution of classes per batch. Mixed Batch effects (Additive + Multiplicative) are then loaded globally (i.e., all variables carry a similar component of batch-correlated effects) onto batch 0 only. This means that data from samples that belongs to batch 0, which we denote as X, will be replaced by Z(X + Y), where Y is the additive factor (arbitrarily denoted as $\sqrt{5}$) and Z is the multiplicative factor (arbitrarily denoted as 1.2). To check whether results are altered because of different batch effects, we repeat all analyses with additive only (i.e., X + Y) and multiplicative only (i.e., Z(X)) batch effects as well (Figs. S1–S3). We then simulate missing data by randomly dropping 50% of the data for each variable (Fig. 1D). Hence, the type of simulated MVs presented in this study are essentially MCAR. We then perform imputation according to the M1, M2, and, when applicable, M3 strategies (Fig. 1E).

In our initial simulation, 4 BECAs (ComBat, BMC, Harman, SVA) were evaluated on batch effect removal efficacy using gPCA (Fig. 1F) and imputation accuracy using RMSE (Fig. 1G). To demonstrate reproducibility, the analysis was repeated 10 times. In our preliminary analyses, the analysis was also repeated 100 times, however, no strong differences were observed due to the relative simplicity of the simulations (Fig. S4).

High-throughput data measuring proteins (proteomics) and genes (genomics) are common in biomedical research and often require both batch correction and MVI.

To demonstrate applicability to proteomics, the analysis was repeated on a renal cell carcinoma (RCC) proteomics dataset^15^. Typically, in a proteomics dataset, the rows represent the proteins and columns represent protein samples. RCC is a benchmark kidney tissue dataset comprising 3 technical replicates (batch effects are induced by combining any 2 replicates together). After omitting all rows with missing values (to measure imputation accuracy, actual data should not have any missing values), we combine the first 2 batches, each batch consisting of 4 protein samples.

To demonstrate applicability to genomics, analysis was repeated on a combined breast cancer genomics dataset, GDS4056 and GDS4057^16^. Typically, in a genomics dataset, the rows represent the genes and columns represent gene samples. GDS4056 and GDS4057 are HER-2 normal breast cancer RNA datasets from different cohorts, comprising of 2 classes: ER-positive and ER-negative subtypes (batch effects are induced by combining samples from the same subtypes but from different datasets together). To obtain a dataset with balanced batch distribution, we combined 32 ER-positive samples from GDS4056 and the first 32 ER-positive samples from GDS4057. Note that there is only one true sample class: ER-positive and 2 batches: GDS4056 and GDS4057, comprising 32 samples each.

To render our results more comparable across evaluations, the RCC dataset and the combined GDS4056/GDS4057 dataset was coerced to have a mean of 5 and standard deviation of 1 (values similar to our initial simulation), while maintaining the original data distribution. This is achieved by z-transforming the RCC dataset by columns, and then adding a fixed value of 5 to all the data. We then followed the pipeline in Fig. 1, where we amplified existing batch effects of the first batch using the additive + multiplicative mixed batch effect approach.

For proteomics and genomics simulations, only 1 BECA (ComBat) is used. For evaluating batch effect, PCA scatterplots are used on top of gPCA. RMSE is used for measurement of imputation accuracy.

#### Power

To investigate the impact of batch correction on class effect, power analysis is conducted. Statistical power is the probability that a hypothesis test will find a statistically significant difference between the 2 classes when an actual difference exists^17^. Therefore, to calculate power, we first perform a t-test on each variable (i.e., row of the data matrix). The null hypothesis of the t-test is that there is no difference between the means of the 2 classes. The t-test is often interpreted by *P* value, defined as the probability of observing results that are equal to or more extreme than what was observed in the data, given that the null hypothesis is true^17^. If the *P* value is larger than the alpha level chosen (e.g., 0.05), any observed difference is assumed to be due to chance, and hence, we fail to reject the null hypothesis in this case. If *P* value is smaller than 0.05, we conclude that there is significant difference between the means of the 2 classes, and hence, we reject the null hypothesis^17^. After calculating the t-test P values for each variable, we calculate the power, which is the number of variables with P value less than 0.05 divided over all variables.

To demonstrate reproducibility of power analysis, we repeated analysis on our genomics dataset (GDS4056/4057) by simulating class effects first, before amplifying the batch effects of the genomics dataset.

#### Precision

To investigate impact on class effects, we compare performance based on Precision, Recall, False Positive Rate (FPR) and False Discovery Rate (FDR). To determine these metrics, we need to obtain the number of True Positive (TP) genes, the number of False Negative (FN) genes, the number of True Negative (TN) genes and the number of False Positive (FP) genes. To achieve this conveniently, we modify our initial simulations such that class effects are loaded for only half of the genes (as opposed to all). The first half of the genes with simulated class effects are classified as positives while the other half with no simulated class effects are negatives. Amongst positive genes, we can obtain the True Positive (TP) genes (i.e., genes with *P* value < 0.05) and False Negative (FN) genes (i.e., genes with *P* value ≥ 0.05). Amongst negative genes, we can obtain the True Negative (TN) genes (i.e., genes with *P* value ≥ 0.05) and False Positive (FP) genes (i.e., genes with *P* value < 0.05).

The formulae for Precision, Recall, FPR and FDR are as follows:

$$\begin{aligned} & Precision = \frac{TP}{{TP + FP}} = 1 - FDR \\ & Recall = \frac{TP}{{TP + FN}} \\ & False\, Positive\, Rate\, \left( {FPR} \right) = \frac{FP}{{FP + TN}} \\ & False\, Discovery\, Rate\, \left( {FDR} \right) = \frac{FP}{{FP + TP}} \\ \end{aligned}$$

## Results

### Simulation labels (briefly)

**Figure 2:**
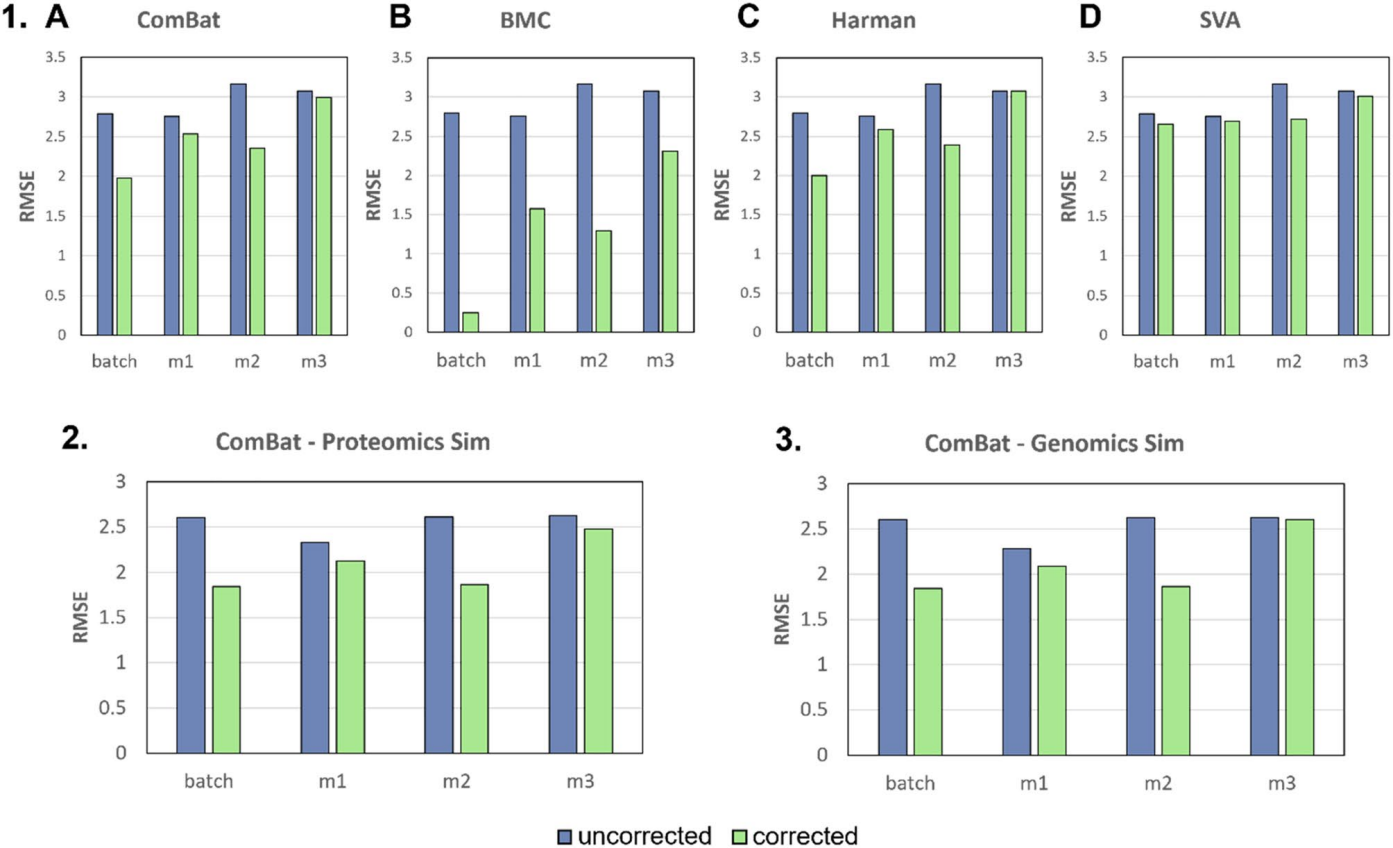
4 Batch effect correction algorithms (BECAs) are used for evaluation of imputation accuracy for **1.** Initial Simulation based on the root mean square error (RMSE): (**A**) ComBat (**B**) BMC (**C**) Harman (**D**) SVA. Only ComBat is used for subsequent evaluation of imputation accuracy for **2.** Proteomics Simulation and **3.** Genomics Simulation based on the root mean square error (RMSE). Lower values indicate higher similarity to original data matrix.

To analyse the impact of MVI on imputation accuracy and global batch effect correction, we compare batch effect correction performance (via RMSE and gPCA delta scores) of BECAs on post MVI data against the original performance of BECAs on data without any missing values. In general, we have the original data (without batch effects), and the modified data with the batch effects. After we correct the modified data with batch effects, we compare it to the original (without batch effects). Therefore, we have controls such as “batch” (i.e., data without missing values but with batch effects) for pre batch corrected imputed data (m1 batch, m2 batch, m3 batch) and “batch corrected” (i.e., data without missing values but with batch effects corrected by BECA) for post batch corrected imputed data (m1 batch corrected, m2 batch corrected, m3 batch corrected). However, before comparing the performance of BECAs on post MVI data versus on data without missing values, it is good to have a gauge on how good the original performance of BECAs is by comparing batch corrected against the true null (i.e., original data with class effects (if any) preserved but no batch effects). RMSE for true null is always 0 and is therefore not necessary to be shown on the RMSE graphs (Fig. 2). Even though our focus is on batch corrected imputed data, it is necessary to have pre batch corrected imputed data as control to investigate if the impact of MVI on batch effects itself matters. This analysis is then extended towards power analysis for investigating the impact on class effects after batch effect correction, which is why the controls and legends for power and t-statistic remain the same for gPCA delta.

### Batch-sensitized missing value imputation improves batch correction

Root mean square error (RMSE) measures the difference between imputed data and true data. The lower the RMSE, the better the imputation accuracy. We first evaluate RMSE across 4 BECAs (ComBat, BMC, Harman and SVA) on mixed batch effects (Fig. 2.1), using the three imputation strategies.

RMSE results show that M2 produces the lowest RMSE across BECAs (except for SVA where batch correction is unexpectedly unstable) (Fig. 2.1). With ComBat, M2 showed a 7.2% and 21.3% improvement in RMSE over M1 and M3 respectively. M2 with Harman showed similar outcomes to ComBat, with 7.4% and 22.2% improvements over M1 and M3 respectively. BMC displayed the greatest benefit from M2, reaching 17.9% and 43.9% reductions in RMSE compared to M1 and M3 respectively. Finally, SVA with M2 showed negligible difference from M1, and only a 9.6% RMSE reduction compared to M3. These results suggests that M2 is generally a superior strategy. However, it is unknown if this observation extends toward real biological data where complex inter-correlations amongst variables exist. Hence, we included real proteomics and genomics data for subsequent analyses.

In addition, we noted that in general, most BECAs (with the exception of SVA) respond favourably to M2. Amongst these, ComBat is the most widely used^18–20^ and also a strong performer across many benchmark evaluations^21^. Our goal is not to identify the best BECA, but rather to demonstrate that batch co-variate sensitization is necessary for effective MVI. And so, for subsequent analyses, we only report results based on ComBat.

In agreement with simulations (Fig. 2.1), M2 provides the best imputation accuracy for both proteomics and genomics data due to having the lowest RMSE (Fig. 2.2,2.3). With more sophisticated MVI methods such as KNN and MICE, we found negligible differences (< 1%) between M1 and M2 (Fig. S12). As the K parameter in our KNN imputation was small, the nearest neighbours selected would naturally derive from the same batch. The principle applies similarly to MICE and thus, M2 and M1 would not see much difference.

### Batch correction

Guided principal components analysis (gPCA) delta is a relative estimation of batch proportion in data. The lower the gPCA delta, the smaller the relative degree of batch-correlated separation in data, and hence, less batch effects (scaled between 0 and 1 regardless of actual batch effect magnitude). Interestingly, although RMSE suggests M2 is the best strategy that yields a more similar matrix to original, gPCA suggests that batch effects are more persistent given M2 (Fig. 3.1). This may be attributed to the high percentage of missing values in our simulations (i.e., 50%). Hence, to check whether gPCA determined batch effects are correlated with the number of missing values, we change the missingness from 10%, 20%, 30% and 40% (and keep the results for 0% and 50% from the initial simulation) for M2. Our hypothesis was proven correct: after batch correction, remnant batch in M2 is attenuated given lower percentages of missing values (Fig. 4). Subsequent analyses based on PCA, data distribution checks post imputations and batch correction revealed that gPCA was misleading—the apparent undetectability of a batch effect was because of batch signal mixing due to M1 and M3 strategies, resulting in noise generation (see Genomics and Proteomics analysis).

**Figure 3:**
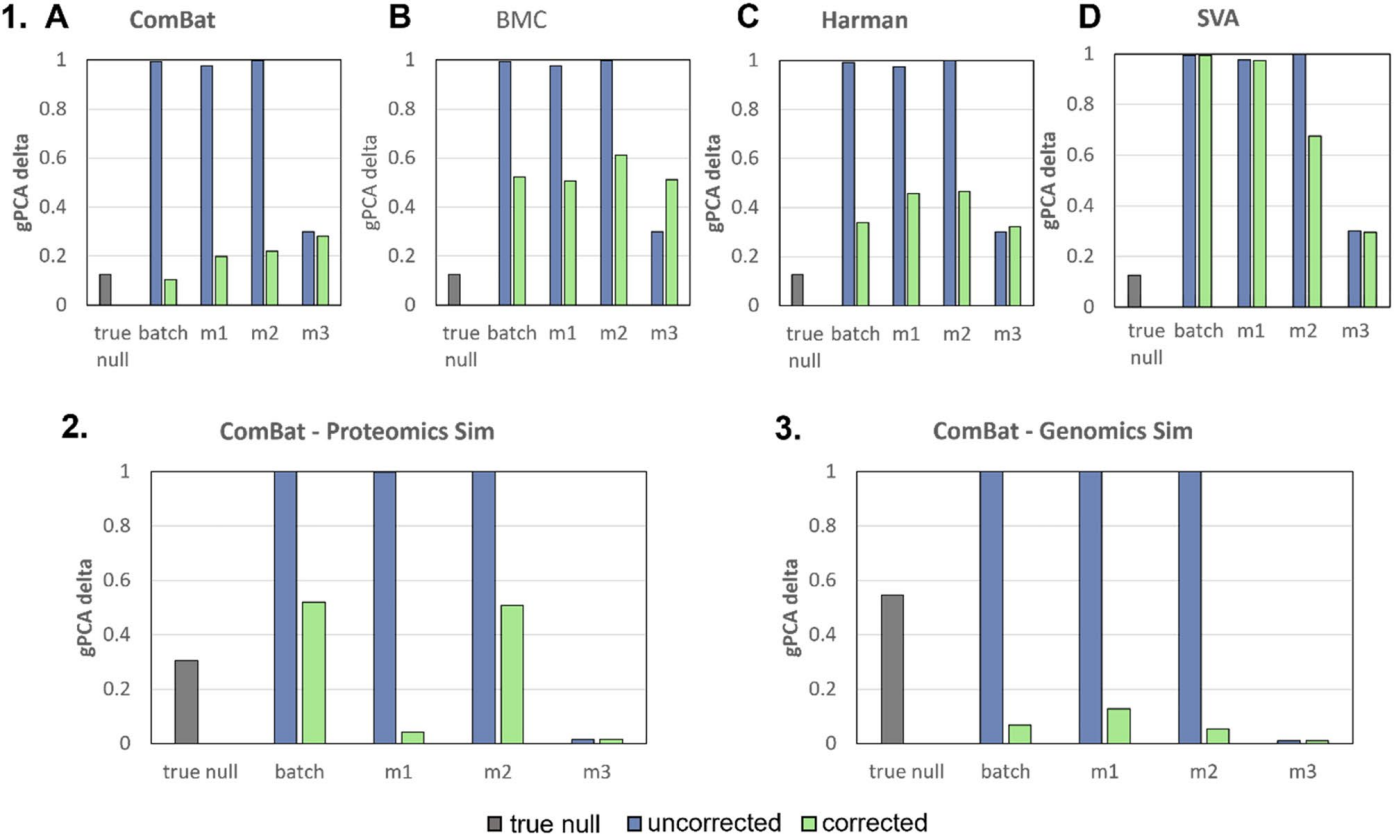
4 Batch effect correction algorithms (BECAs) are used for evaluation of batch correction for **1.** Initial Simulation based on the gPCA delta: (**A**) ComBat (**B**) BMC (**C**) Harman (**D**) SVA. Only ComBat is used for evaluation of batch correction for **2.** Proteomics Simulation and **3.** Genomics Simulation based on the gPCA delta. Lower values indicate less batch-correlated separation in data.

**Figure 4:**
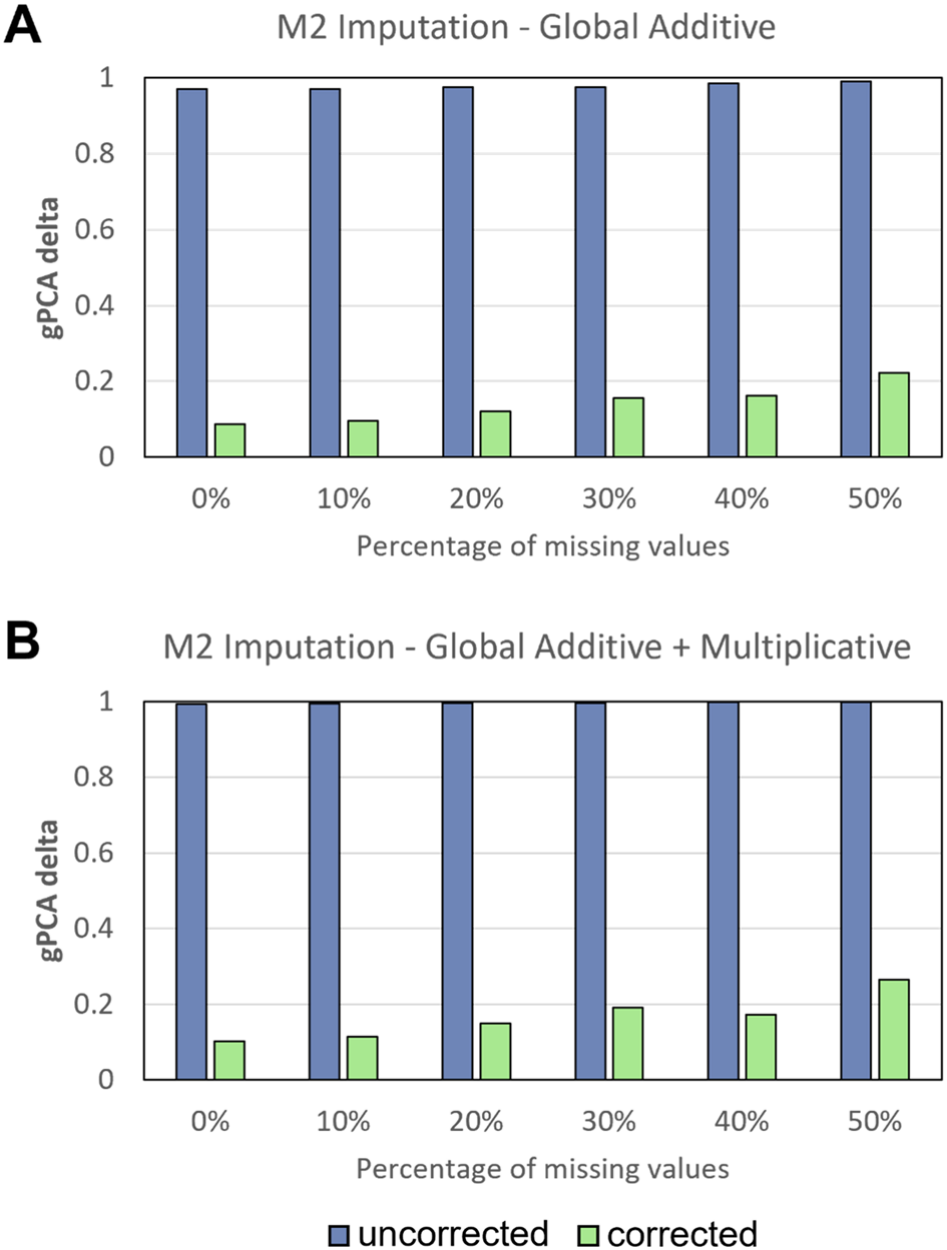
Initial simulation, with varying % of missing values (i.e., 10%, 20%, 30%, 40%, 50%) imputed by M2 only, is used for evaluation of batch correction for M2 based on gPCA delta. Only ComBat is used as BECA for this evaluation. gPCA delta results for both (**A**) additive only and (**B**) mixed batch effects (Additive + Multiplicative) scenarios showed that after batch correction, remnant batch in M2 is attenuated given lower % missing values.

**Figure 5:**
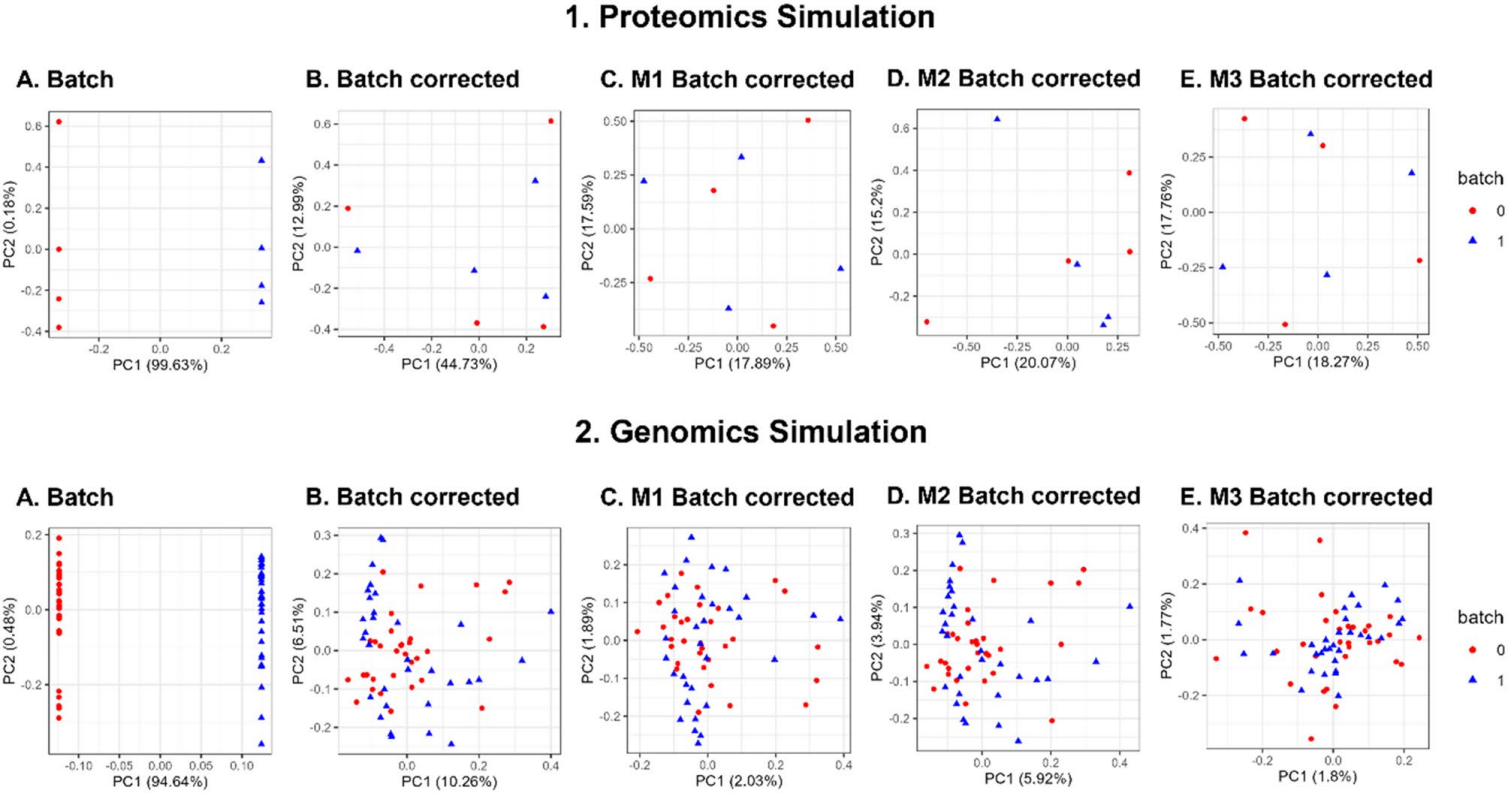
PCA Scatterplots for **1.** Proteomics Simulation and **2.** Genomics Simulation showed that despite reporting higher gPCA levels for M2, samples appear well-mixed, with no apparent batch effects for all imputation strategies (M1–M3), given the first two principal components (PC1 and PC2) (c.f. Figure S5 for full version).

Similar to pure simulations (Fig. 3.1), M2 does not perform well for proteomics based on batch correction given gPCA delta (Fig. 3.2). Unlike simulations and proteomics, M2 performed on genomics data has somewhat good batch effect reduction (i.e., lowest gPCA delta), however, it is still quite comparable to M1 (Fig. 3.3). We found this highly suspicious as M1 and M3 are reporting much lower gPCA deltas than expected while also exhibiting high dissimilarities to the original matrix (high RMSE). To unravel this, we checked the principal components analysis (PCA) scatterplots based on the first 2 principal components (PCs) (Fig. 5).

Given the first 2 PCs, batch effects across M1 to M3 appears well resolved for both proteomics (Fig. 5.1C–E) and genomics (Fig. 5.2C–E). Despite reporting higher gPCA levels for M2, samples appear well-mixed with no apparent batch effects for all imputation strategies (M1–M3) given the first two principal components (PC1 and PC2). We do note that for M2, some same-batch samples do appear more clustered following batch correction (Fig. 5.1D).

We additionally assessed the gPCA delta using KNN and MICE for M1 and M2. As with the RMSE results, gPCA delta differed only slightly between both scenarios (Fig. S12).

### M1 and M3 inflates intra-sample variance

**Figure 6:**
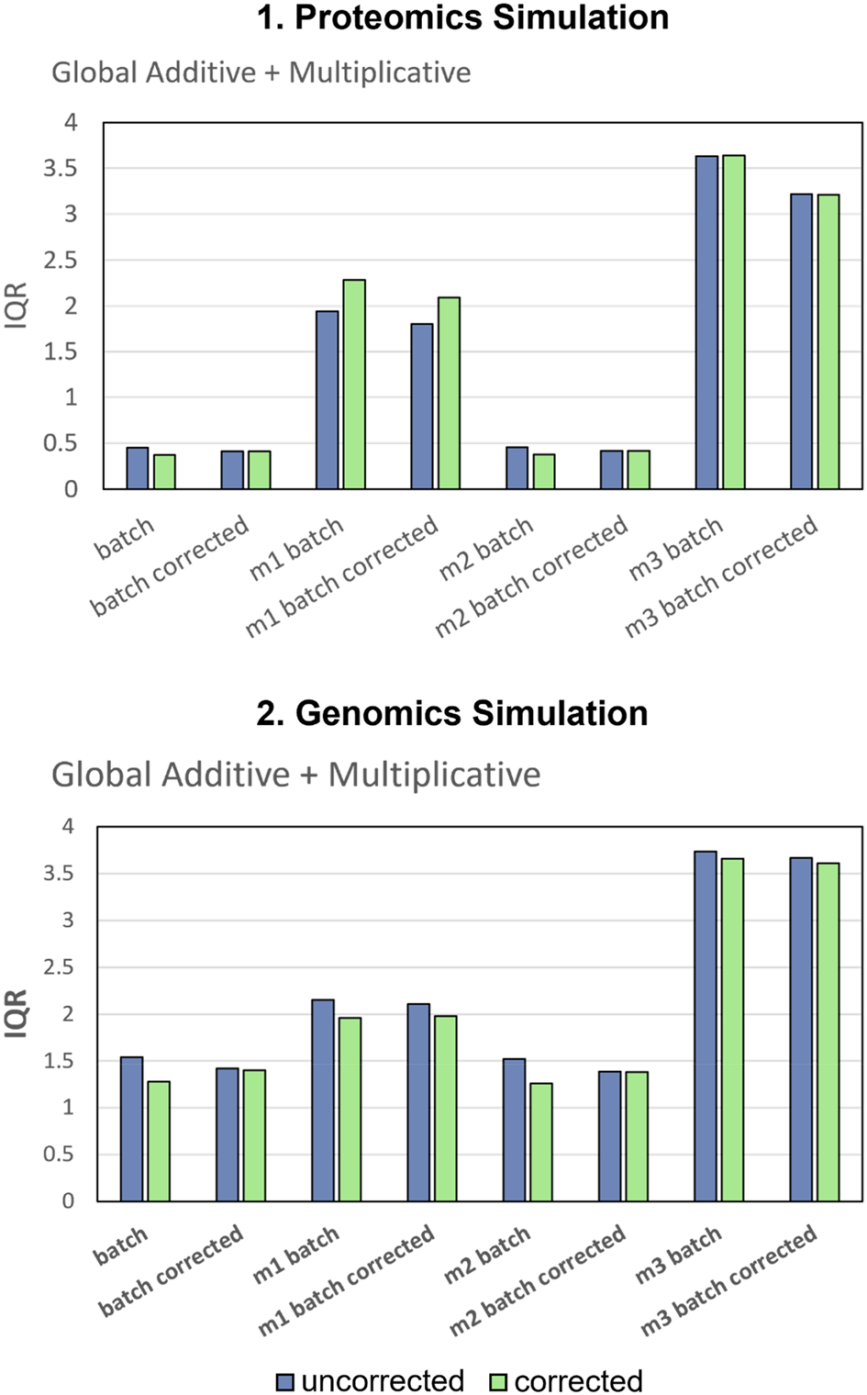
Interquartile Range (IQR) for **1.** Proteomics Simulation and **2.** Genomics Simulation showed that while M2 preserved similar sample variances to original data, M1 and M3 variances were grossly inflated.

Batch-corrected M1–M3 samples appear fairly well-mixed given PCA analysis. It is possible that gPCA may be misreporting the extent of residual batch effects. However, this does not explain the high RMSE observed in M1 and M3 or the low RMSE given M2. Hence, we devised a simple approach to check sample distributions in original data, and also given M1–M3 imputation strategies. We expect high variance in samples following M1 and M3 imputation, thereby giving rise to high RMSE. Our hypothesis was proven correct: while M2 preserved similar sample variances to original data, M1 and M3 variances were grossly inflated (Fig. 6). To us, this means that M1 and M3 imputation strategies have traded batch effects for noise. This explains the high RMSE and low gPCA observations.

### Power and effect size

**Figure 7:**
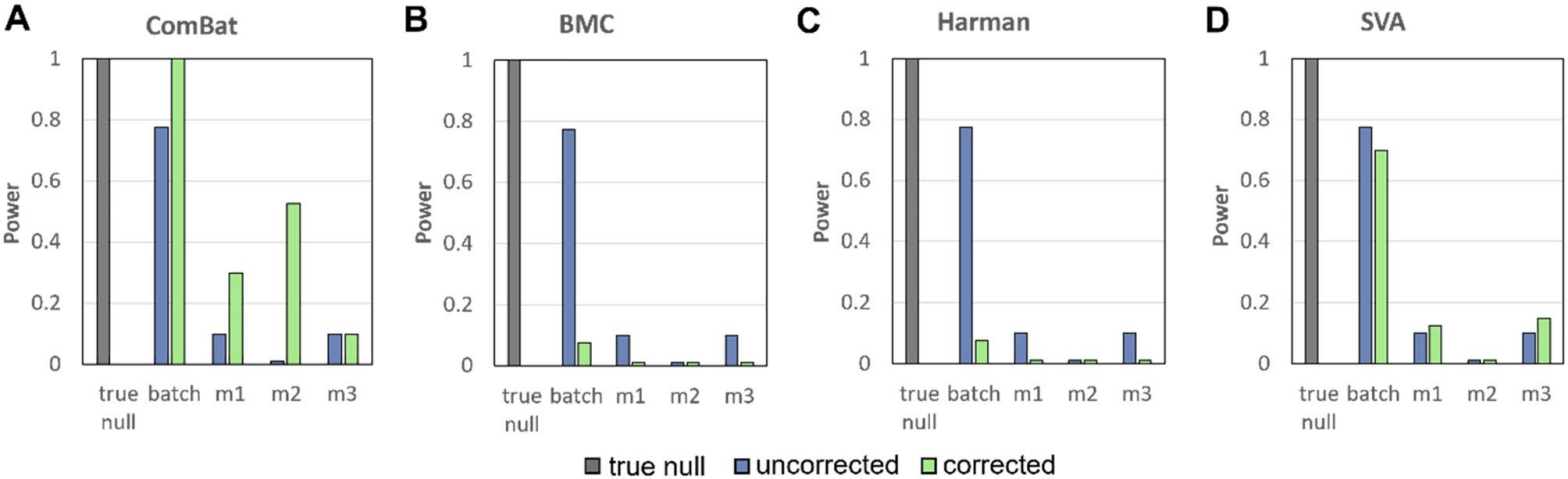
4 Batch effect correction algorithms (BECAs) are used for evaluation of power for Initial Simulation based on statistical feature selection: (**A**) ComBat (**B**) BMC (**C**) Harman (**D**) SVA. Higher values indicate better performance (higher recall of correct features).

Amongst the 3 imputation strategies, M2 provides the highest statistical power (i.e., the highest probability of detecting class effects when it does exist) after batch correction. However, we noted (and with good reason; see Discussion), it will never be as high as original batch corrected data with no missing values (with the exception of BMC) (Fig. 7). This suggests that imputation strategies are imperfect, and information loss occurs anyway.

Nonetheless, we do note that for genomics simulation in particular, statistical power for M2 after batch correction is cross comparable to the original batch corrected data (Table 1). We hypothesize that this is because sample size of genomics data is large enough such that M2 is able to make meaningful imputations. This intuition turns out to be correct as after reducing the genomics data to a 20 × 20 matrix (similar to our pure simulation dimensions), M2 batch corrected no longer performs as well as batch corrected (Fig. S11).

**Table 1:** Power for genomics simulation.

Variable	Power (2 d.p.)
Base power	1.00
Batch	1.00
Batch corrected	1.00
m1 Batch	0.96
m1 Batch corrected	0.99
m2 Batch	0.84
m2 Batch corrected	1.00
m3 Batch	0.70
m3 Batch corrected	0.70

Only ComBat is used for evaluation of power for Genomics Simulation based on statistical feature selection. Higher values indicate better performance (higher recall of correct features).

**Figure 8:**
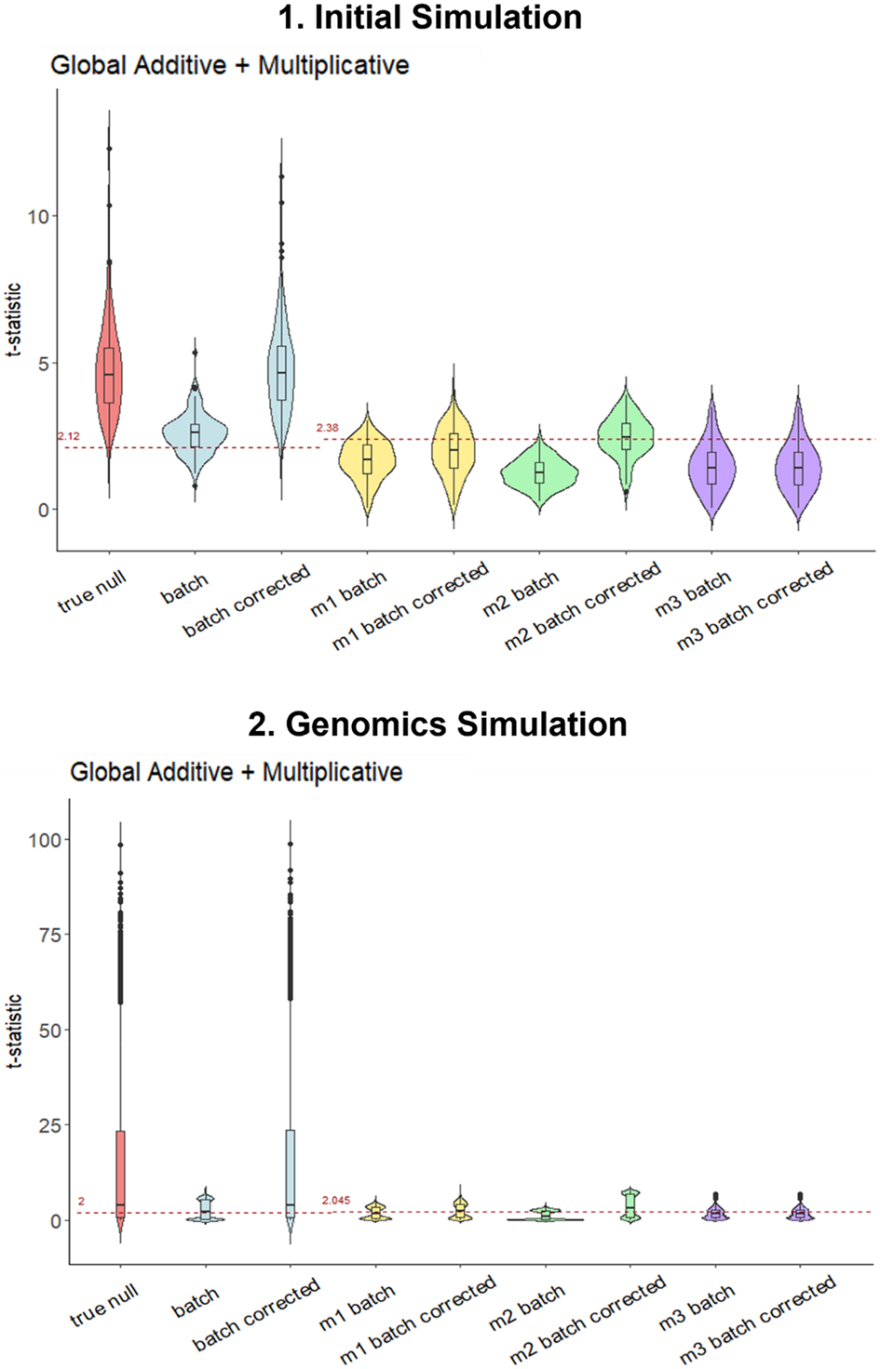
t-statistic distributions for **1.** Initial Simulation and **2.** Genomics Simulation reveal that although power is the best for M2, all imputation strategies (M1–M3) suffer from a reduction in effect size.

Although M2 provides the best power, we hypothesize that imputation can dilute class effects as there are in fact, two classes within each batch. This intuition turns out to be correct as the t-statistics associated with M1, M2, M3 are generally lower than the original (without missing values) (Figs. 8 and S7).

We also noted (Fig. 4) that BMC and SVA do not work in these simulations where power is negatively affected even in original data. BMC and SVA do not work well on multiplicative-only and additive-only batch effect scenarios either (Fig. S3). As neither methods are often reported as optimal in literature, nor are they able to satisfactory restore pre-batch performances in original data, we do not consider these for further analyses on real data.

One may expect therefore, that imputation based on the same class and batch may yield better results (we term this approach M2.1; see Fig. S9). However, we find that this was not exclusively so. While M2.1 did indeed yield t-statistics more similar to original data, it did not outperform M2 in terms of RMSE, gPCA and power.

### Precision, recall, false positive rate (FPR), false discovery rate (FDR)

**Figure 9:**
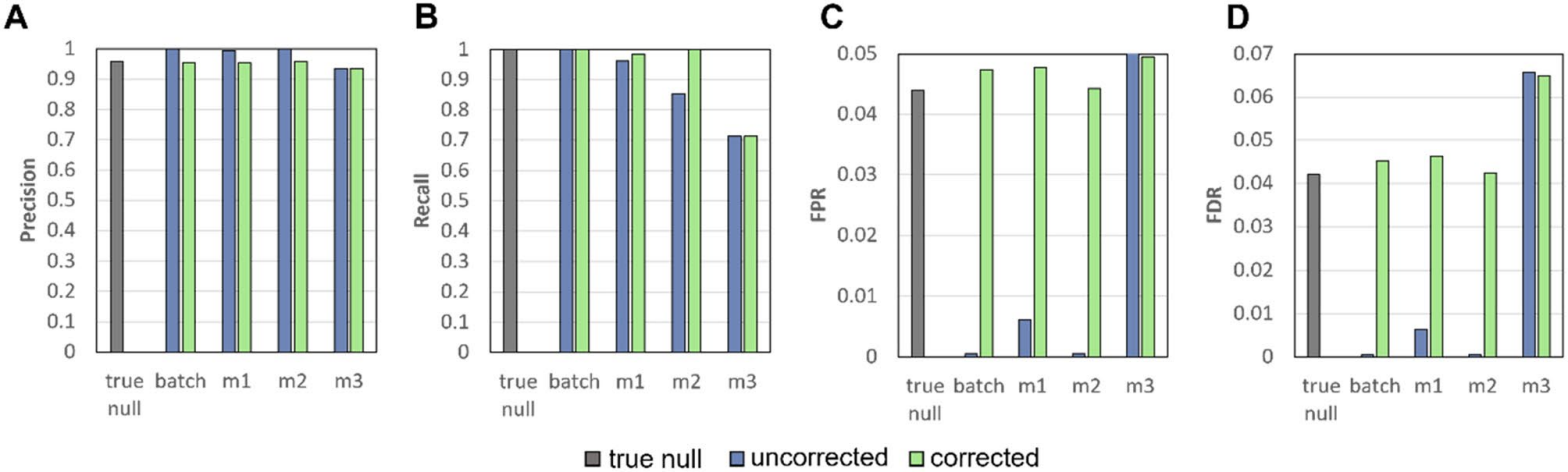
Performance Metrics for Genomics Simulation reveal that M2 performs the best among post MVI data. Higher values indicate better performance: (**A**) Precision (**B**). Recall. Lower values indicate better performance: (**C**) False Positive Rate (FPR) (**D**) False Discovery Rate (FDR).

Power deals with false negatives. However, we also need to consider mistakes stemming from false positives. We evaluate performances based on precision (proportion of correct features amongst selected features) and recall (proportion of correct features amongst all correct features), and also the false positive rate (FPR; the proportion of false positives stemming from wrong features) and false discovery rate (FDR; the proportion of wrong features amongst selected features). The results show that after batch correction, M2 performs the best with highest precision and recall and lowest FPR and FDR among post MVI data. What is especially notable is that M2 batch corrected seems to perform quite comparably to batch correction (i.e., data without missing values) (Fig. 9). This is because sample size of genomics data is large enough for M2 to make meaningful imputations (same reasoning as to why, for genomics simulations, statistical power for M2 after batch correction is comparable to the original batch corrected data; see Results: Power and Effect Size).

In the KNN and MICE simulations, these metrics did not improve in M2 scenarios (Figs. S13 and S14). This is not completely unexpected, as we know that these complex methods tend to introduce some biases which can negatively affect downstream analyses.

## Discussions

### RMSE is more informative than gPCA delta

Although it does not estimate batch effects directly, RMSE (provided we have the original data) is a more informative measure than gPCA delta. gPCA delta lacks sensitivity and fails when batch effects are diluted (Fig. 3). In our analyses, M1 and M3 approaches report lower batch effects than M2 even though there is a concomitant increase in intra-sample variances. If we were to believe gPCA alone, then we would have been misled into thinking M1 and M3 are reasonable imputation strategies.

In our simulations, we are able to measure imputation accuracy via RMSE, and so, we can compare how similar the imputed matrix is to the original. RMSE suggests that in spite of lower reported gPCA delta, M1 and M3 imputed matrices are distinct from original data (with no MVs). In contrast, M2 imputed matrices are comparatively similar to original data (Fig. 2).

Unfortunately, on real data, there is no original data reference, and so RMSE cannot be used. However, our first recommendation is that estimates of batch effects, whether summary (gPCA delta) or visual (PCA scatterplots), should be considered carefully: The lack of apparent batch effects in data, does not mean data quality is pristine.

### M1 and M3 trades batch effects for noise and this conversion is irreversible

While batch effects appear to be “mitigated” (Fig. 6), M1 and M3 result in increased noise (i.e., larger interquartile range) in the data (Figs. 7 and S10). Hence, it is sensible to impute missing values by M2 to avoid introducing additional noise into the data.

An important observation is that intra-sample variance increases dramatically in M1 and M3 imputed samples, particularly so for proteomics data (Fig. 7). This increased variance does not decrease post-batch correction, meaning it is no longer recognized as a batch effect, and so is not removed. In a practical setting, this also means that should you obtain the imputed matrix generated under wrong assumptions (e.g., did not consider the batch co-variate), it is too late to apply a batch correction algorithm. This is an important example why data pre-processing considerations are important.

### Should we use both batch and class sensitization strategies together?

The value of the t-statistic and accompanying degrees-of-freedom (dependent on the actual number of non-missing values) determines the statistical *p*-value. In general, the t-statistics of M1, M2, M3 are lower than batch corrected (Figs. 8 and S7). The t-statistics indicate a drop in effect size estimations for all imputation strategies (M1, M2, M3). Therefore, even though M2 gives the highest power for post MVI data after batch correction (Fig. 4), the t-statistic distribution is appreciably different between imputed and original data.

The t-test can be divided into 2 components—the numerator is expressed as the difference of means, which is a direct proxy for effect size. The denominator expresses uncertainty on this estimation of effect size and is related to the combined variances amongst samples. By dissecting both the numerator and denominator, it appears that the chief contributor towards reduction in the test-statistic, is due to increased variance due to imputation strategy (Fig. S8).

We considered a more elaborate strategy incorporating both class and batch co-variates. While this strategy did improve the t-statistic distribution, it did not perform as well as M2 in terms of RMSE, gPCA and power.

### Imputation methods are no substitute for complete data

In spite of improved performances given more reasonable imputation assumptions, the performance of imputed matrices does not come close to original data without missing values (Figs. 4 and S11). However, we do recognize that we are considering a rather drastic scenario with 50% data loss. However, such high data losses are not unheard of in biological scenarios. In proteomics, 20–30% missing values is usual^22^. In genomics data, completion is normally higher, although missing values do occur, especially at low abundance levels (this is a form of MNAR). If missing values aggregate mostly at low abundance levels with high coefficient of variances, imputation may not provide satisfactory outcomes. Some normalization strategies e.g. gene fuzzy scoring (GFS) advocate ignoring noise from lower abundance levels due to higher noise^23^.

### Limitations and future work

This work illustrates the importance of batch-sensitization in MVI data processing. We often forget use-case limitations in the design of data processing techniques. Another related example is the necessity for class-sensitization in widely used normalization methods such as quantile normalization^24^.

Despite the simplicity of our simulations, the results are consistent. We did not perform probabilistic or random value imputation as these methods also can add noise and unpredictability into our models. It will take more simulations for the results to converge, without adding much value.

Future work may take into account the mechanism of distinct MVs such as MAR and MNAR, which are known to impact downstream analyses^4^. Amongst MVIs, we have opted for the simplest mean-based imputation method which is purely univariate. While global and other more MVI sophisticated methods are not explored exhaustively, we expect that these approaches should also be compatible with M2. The difference was not obvious in our preliminary analyses with more sophisticated MVI methods.. This may be due to the algorithm imputing MVs by drawing information from the most similar data points and thus, drawing information from their own batches anyway, resulting in the diluted effect of M2 imputation. Indeed, some MVIs may already be batch-resistant due to design and assumptions: Identifying which are, explaining why this is so, and determining how robust is the batch resistance, is worth investigating in future works. We also did not perform probabilistic or random value imputation. These methods also can add noise and unpredictability into our models. Presumably, it will take more simulations for the results to converge, without adding much value.

## Conclusions

Missing data is pervasive in real world data. Direct imputation in the presence of batch effects may mislead. We show that M2 approaches (batch-sensitized) improves performance. We also show that imputation by M1 (global) and M3 (cross-batch) introduce noise, which contribute directly towards false positives and false negatives. Indeed, M1 reflects standard practice where batch correction is only considered and performed following global imputation. However, M2 reveals to us that batch effects should be considered early on during the MVI process itself.

## Supplementary Information

Supplementary Information.

## Data Availability

The breast cancer genomics datasets analysed during the current study are available in GEO, [IDs: GDS4056 and GDS4057]. The RCC proteomics dataset is available in PRIDE via accession PXD000672.
