# Supplementary material for: The importance of batch sensitization in missing value imputation
Source: Sci Rep. 2023 Feb 21;13:3003. doi: 10.1038/s41598-023-30084-2 (PMC9944322; doi:10.1038/s41598-023-30084-2)
Supplement: Supplementary file 1 — Supplementary Information. [file 41598_2023_30084_MOESM1_ESM.docx]

# Supplementary Materials

## Supplementary Methods

| **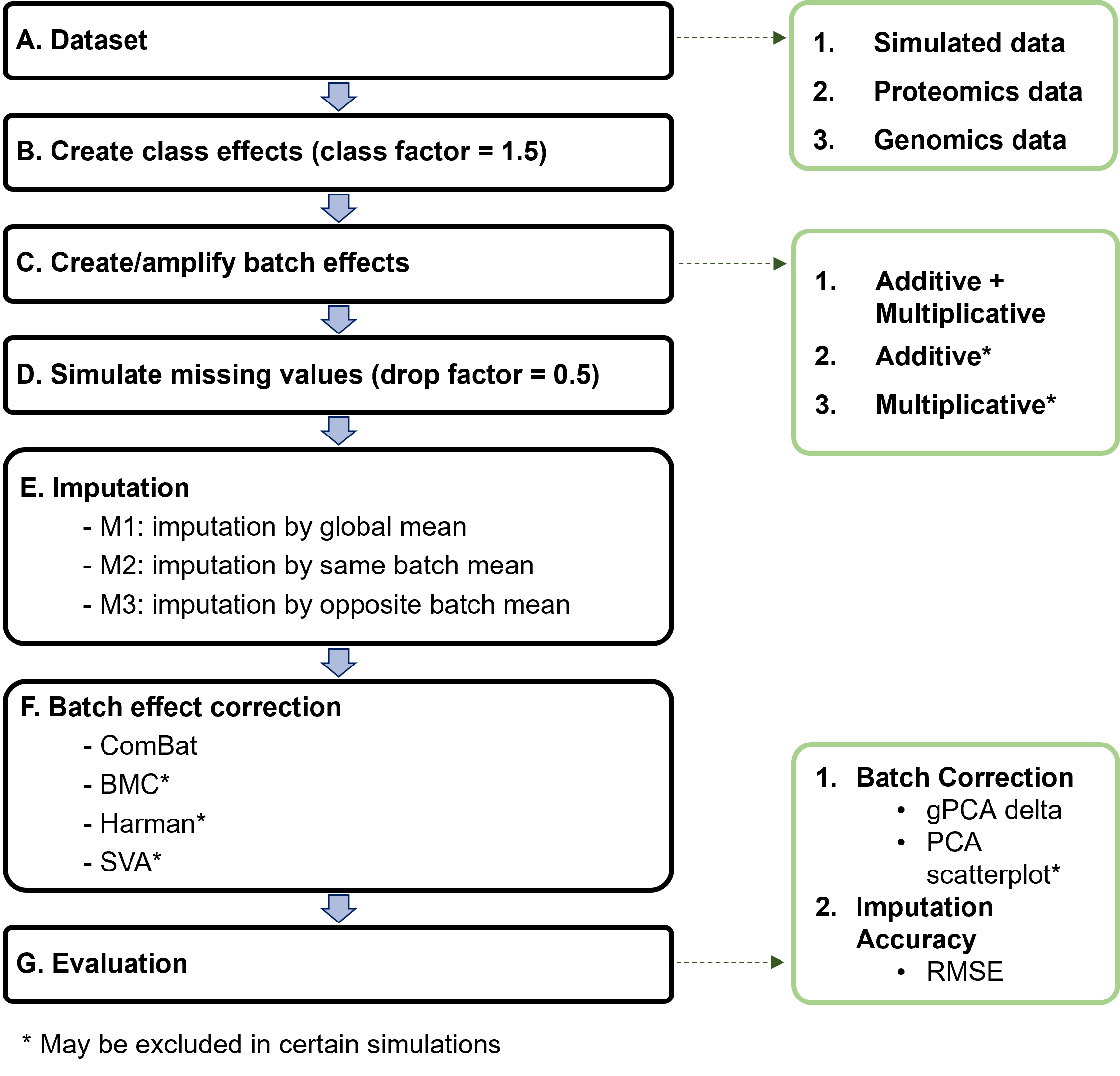**  Figure 1. **1.** Initial Simulation Pipeline **A.** Simulated dataset with normally distributed random numbers **B.** Simulated class effects **C.** Simulated batch effects **D.** Introduction of 50% missing values **E.** Imputation of missing values. 3 sub-conditions for the mean-based averaging imputation method: Global mean imputation (M1), Same batch mean imputation (M2), Opposite batch mean imputation (M3) **F.** Perform batch correction. 4 types of BECAs are used: ComBat, BMC, Harman, SVA **G.** Evaluate the outcome based on the estimation of remnant batch effects and imputation accuracy |
| --- |

## Sophisticated MVI Methods

**k-nearest neighbour (KNN)**

KNN is a very common and simple machine learning MVI method which imputes MVs based on the average of the k nearest data points, determined by Euclidean distance. The idea is that features (e.g., proteins) that are closer are more alike, and thus have similar values. The KNN algorithm used in this study was from the “impute” package (version 1.62.0) in R^13^, with the default k=5. In this algorithm, when missing proportions are more than 50% by row or 80% by column, MVs are replaced with the global mean of the dataset. However, as we simulate MVs in this study by dropping 50% of observations in each row, this will not have a large effect on our dataset. Additionally, as M3 (opposite batch imputation) cannot be performed using KNN, we will only evaluate it using M1 (global imputation) and M2 (same batch imputation) scenarios

**Multivariate imputation by chained equations (MICE)**

MICE is a multiple imputation strategy, which performs multiple iterations of imputations until an explicit value is obtained for each MV. It works by using a placeholder value for the first iteration, then putting it through a linear regression to obtain a new imputed value which will be used in the next iteration. When using MICE, the MVs are expected to be MAR, as the imputations are dependent on information from the observed values. For this study, we used the “mice” package (version 3.14.0) in R^14^. Much like KNN, MICE can only be carried out on M1 and M2 scenarios.

## Supplementary Figures

**
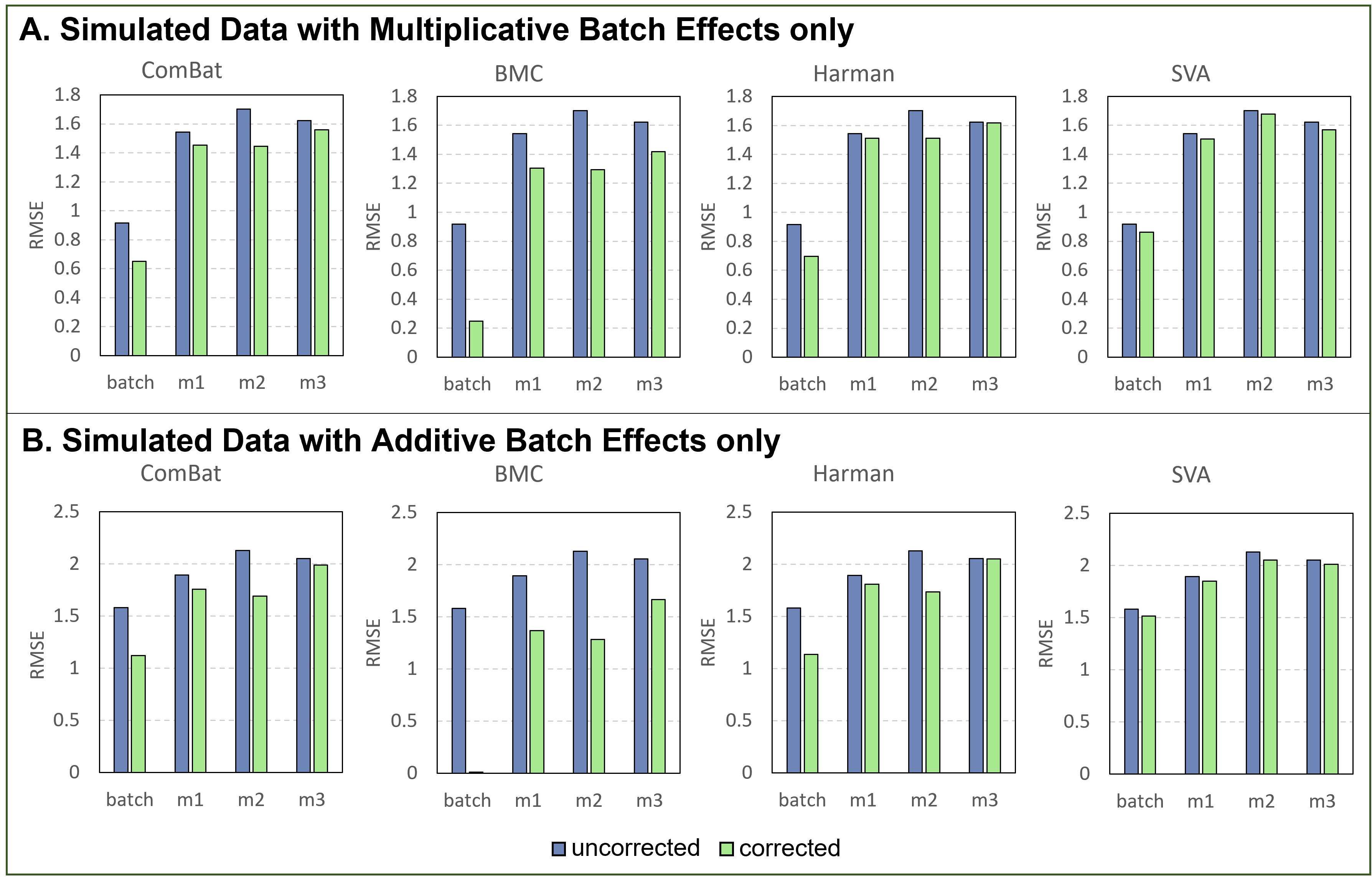
Figure S1.** RMSE for Initial Simulations with **A.** multiplicative only and **B.** additive only batch effects have no strong differences with results with mixed batch effects (Additive + Multiplicative).


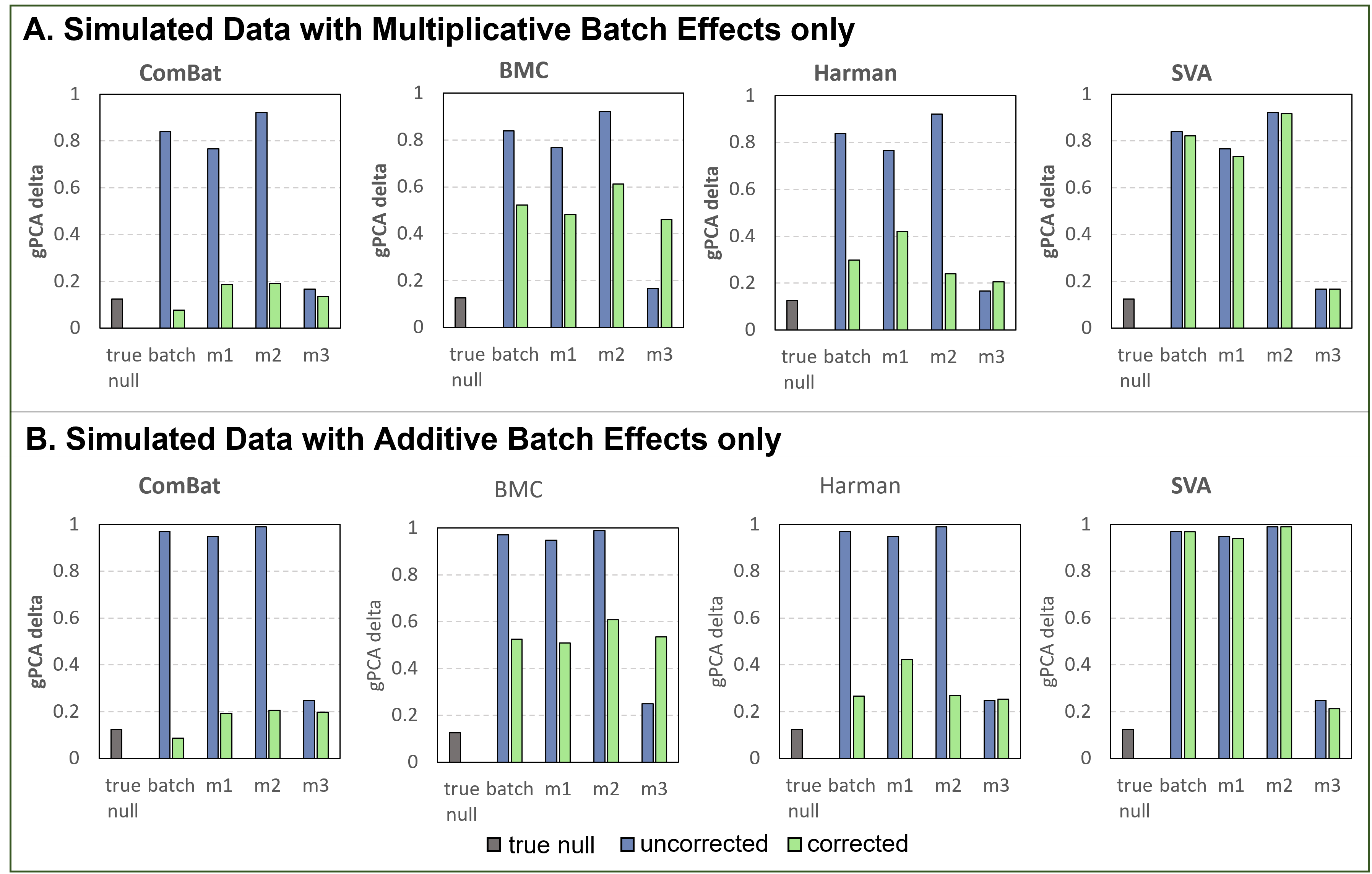


**Figure S2.** gPCA delta results for Initial Simulations with **A.** multiplicative only and **B.** additive only batch effects have no strong differences with results with mixed batch effects (Additive + Multiplicative).


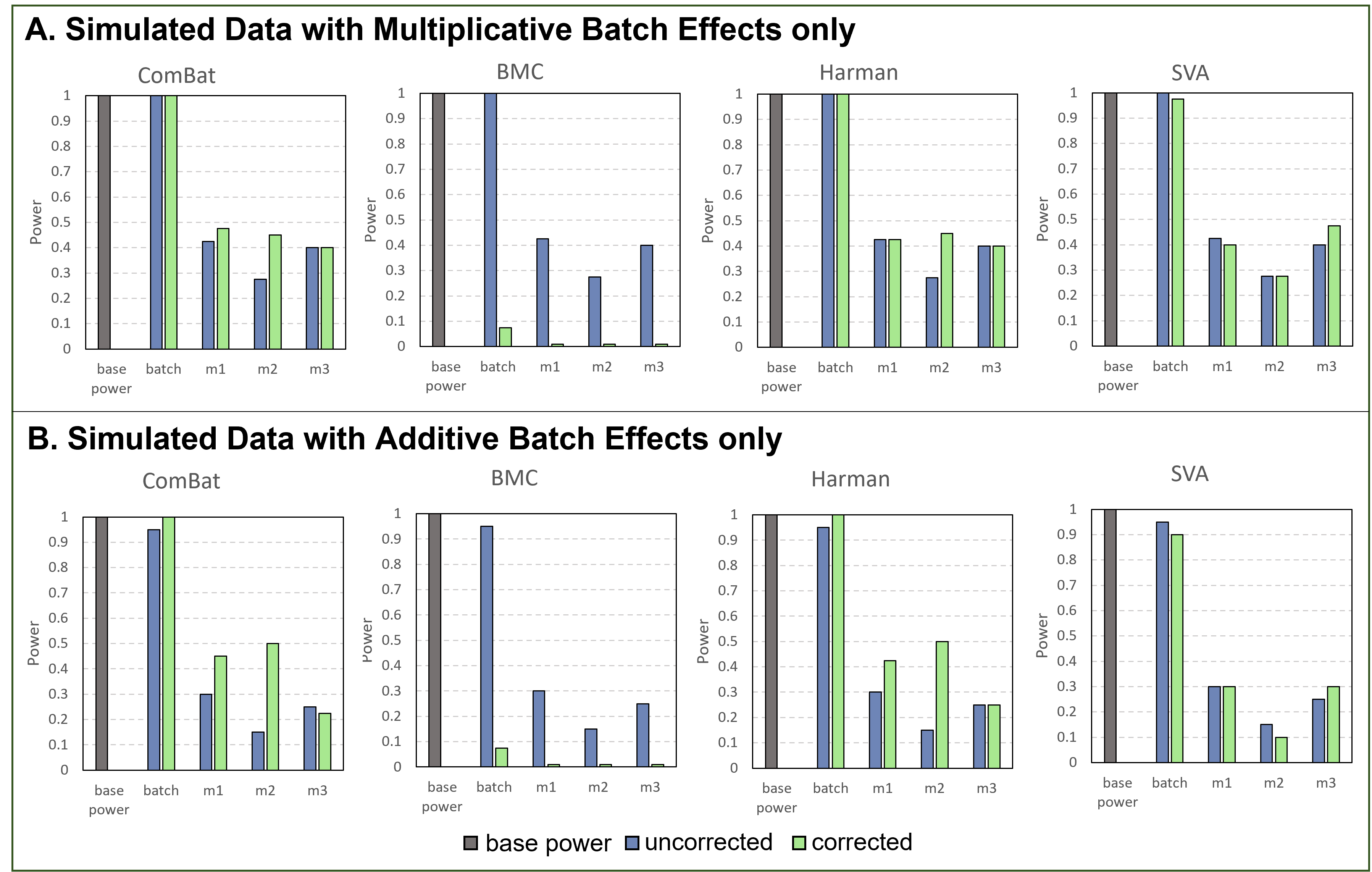


**Figure S3.** Power for Initial Simulations with **A.** multiplicative only and **B.** additive only batch effects show that BMC and SVA do not work well in these scenarios either.

**
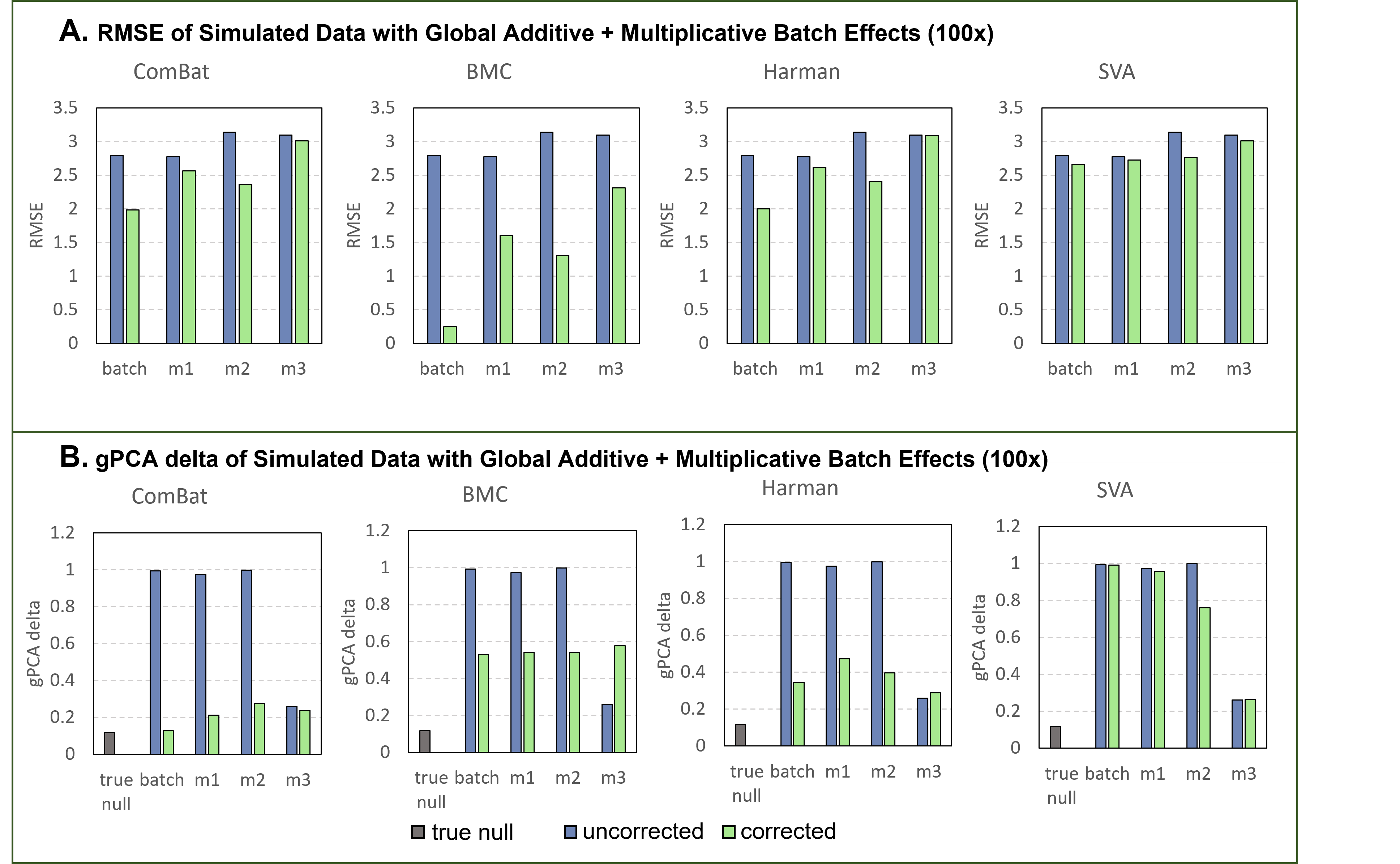
Figure S4. A.** RMSE, **B.** gPCA results for Initial Simulation with 100x repeat have no strong differences with results for 10x repeat.


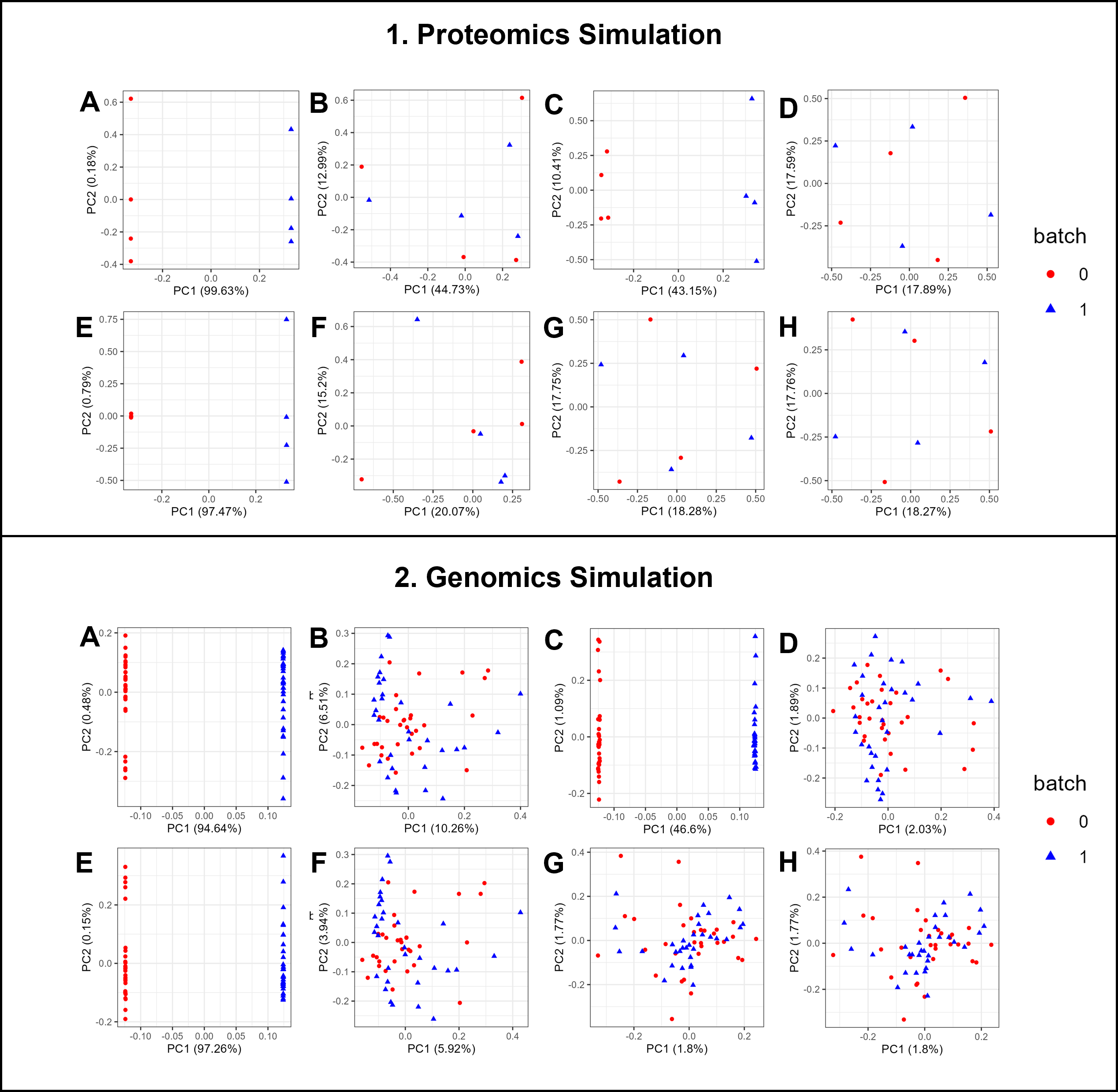


**Figure S5.** PCA Scatterplots (which includes both pre batch corrected and post batch corrected data) for **1.** Proteomics Simulation and **2.** Genomics Simulation showed that despite reporting higher gPCA levels for M2, samples appear well-mixed, with no apparent batch effects for all imputation strategies (M1 to M3), given the first two principal components (PC1 and PC2).

**
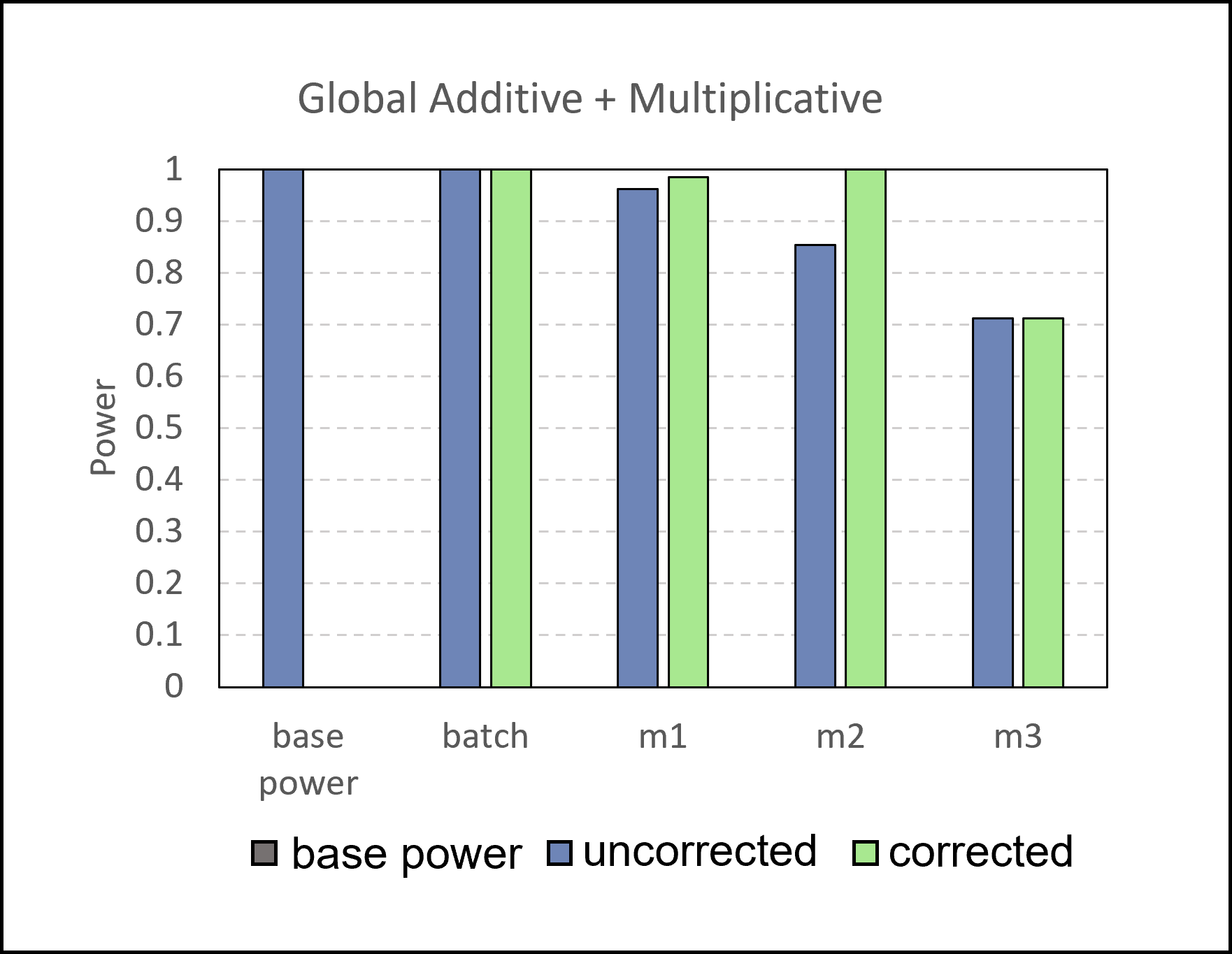
**

**Figure S6.** Only ComBat is used for evaluation of power for Genomics Simulation based on statistical feature selection. Higher values indicate better performance (higher recall of correct features).


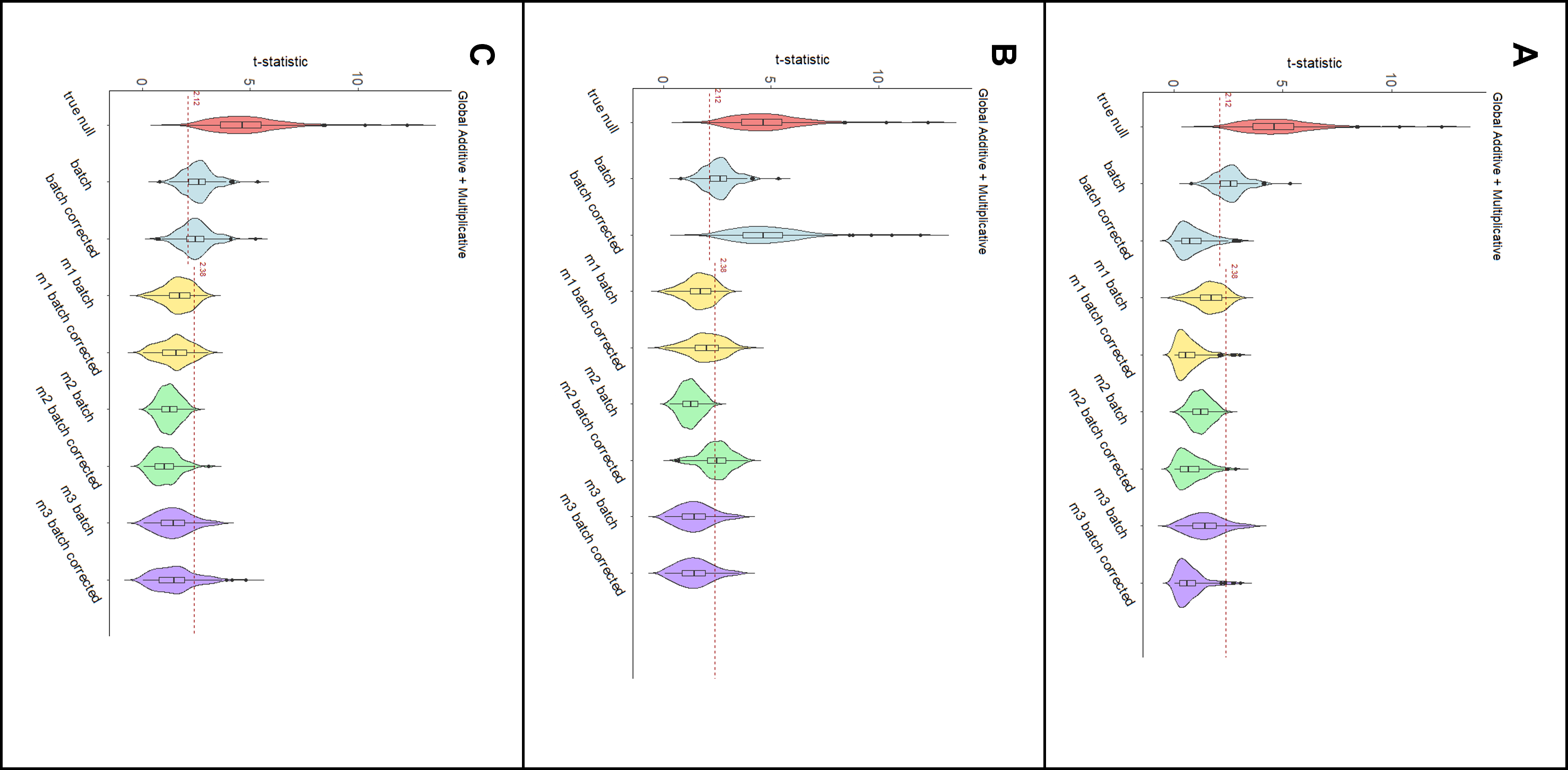


**Figure S7.** t-statistics distribution for Initial Simulation using BECAs such as **A.** BMC, **B.** Harman, **C.** SVA also show that all imputation strategies (M1 to M3) suffer from a reduction in effect size.

**
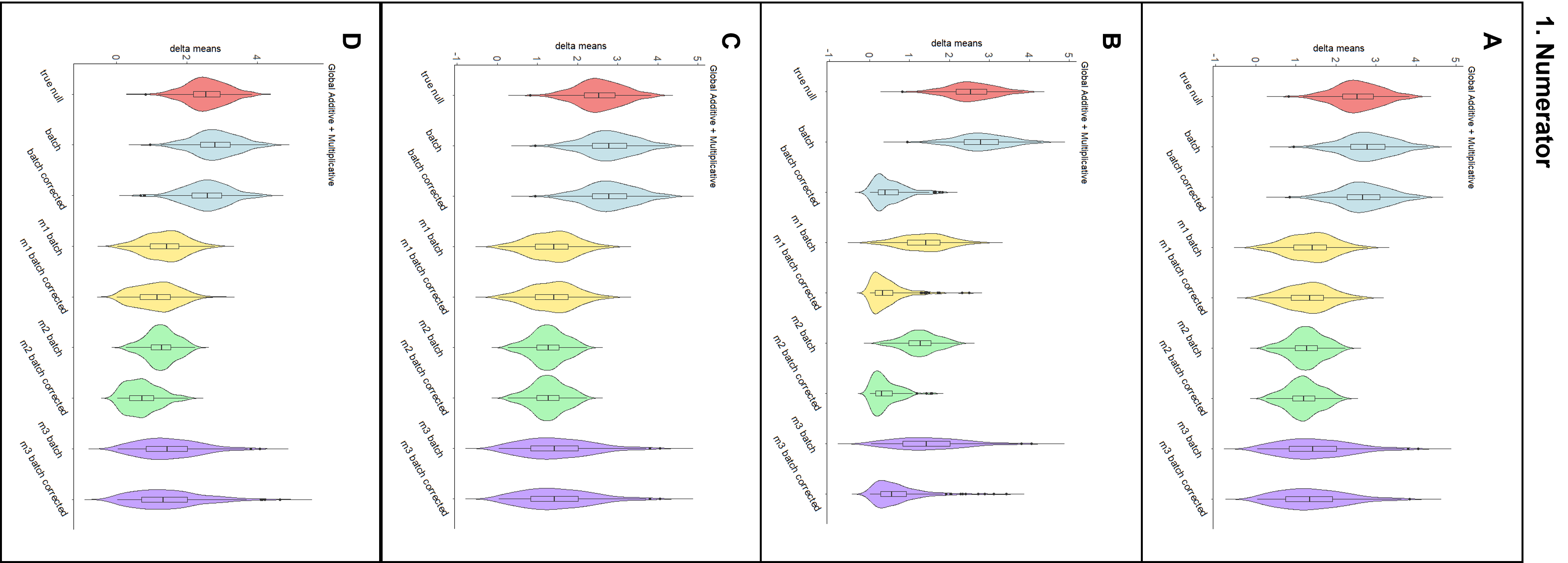

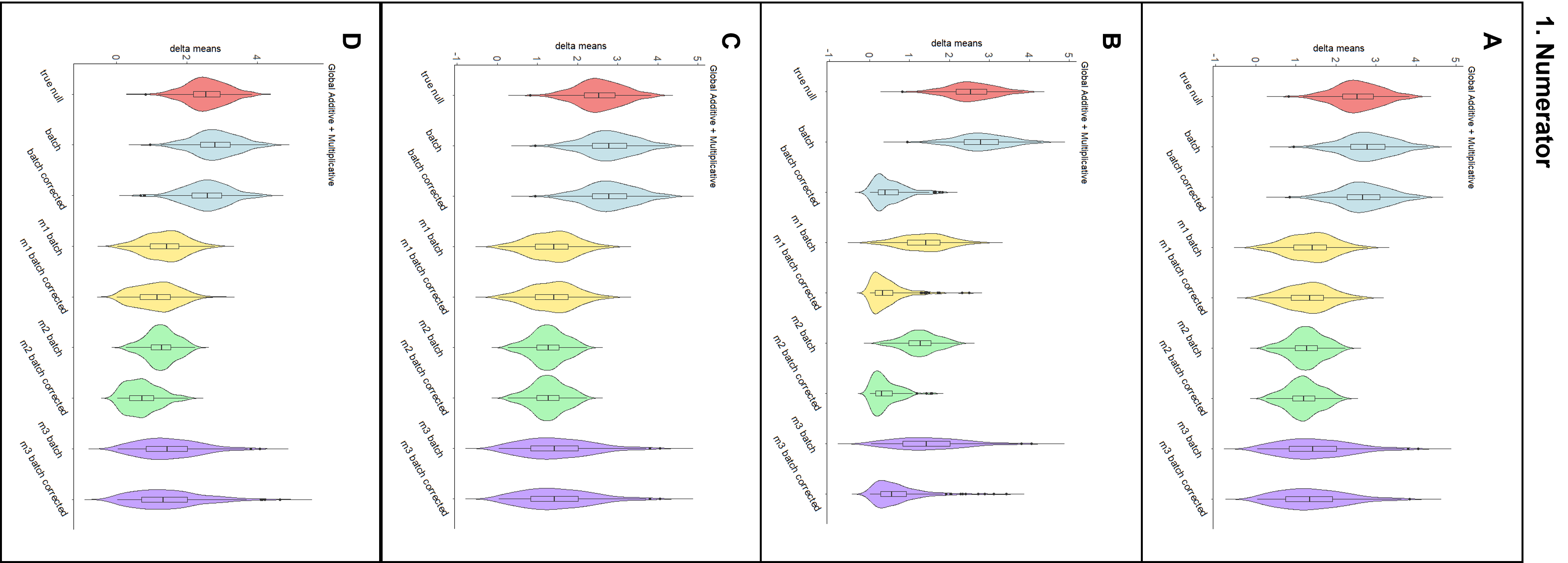

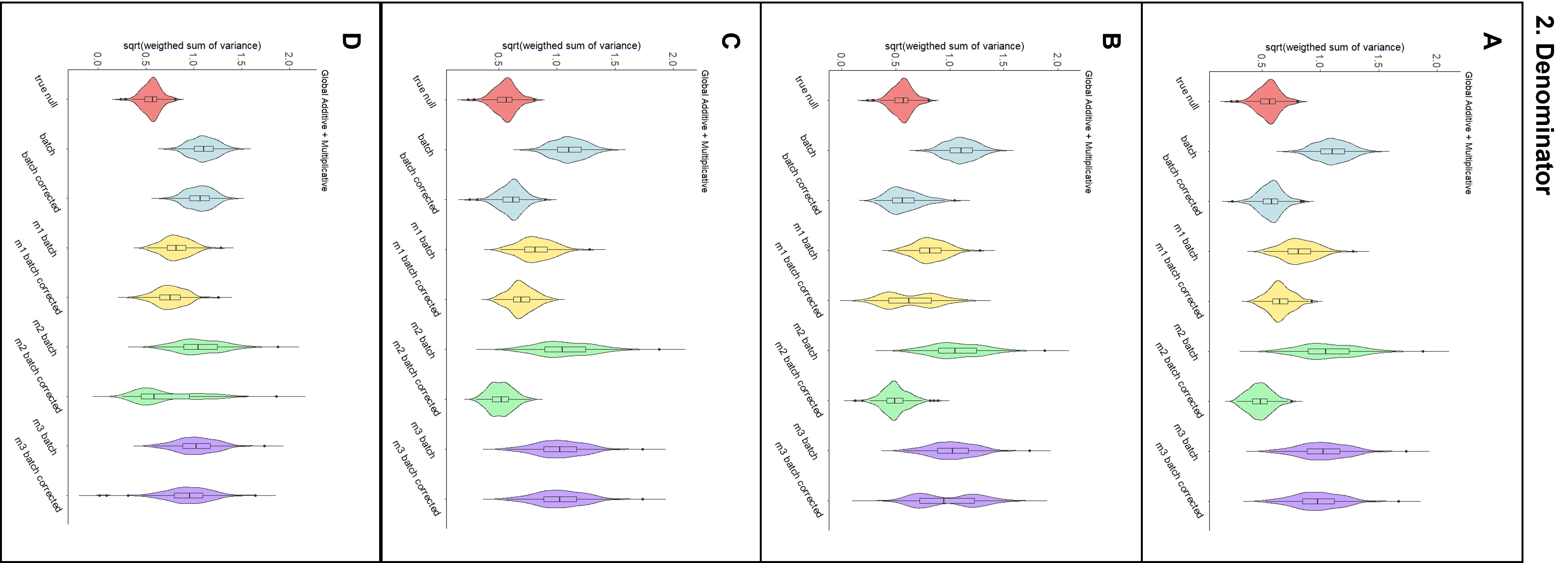
**

**
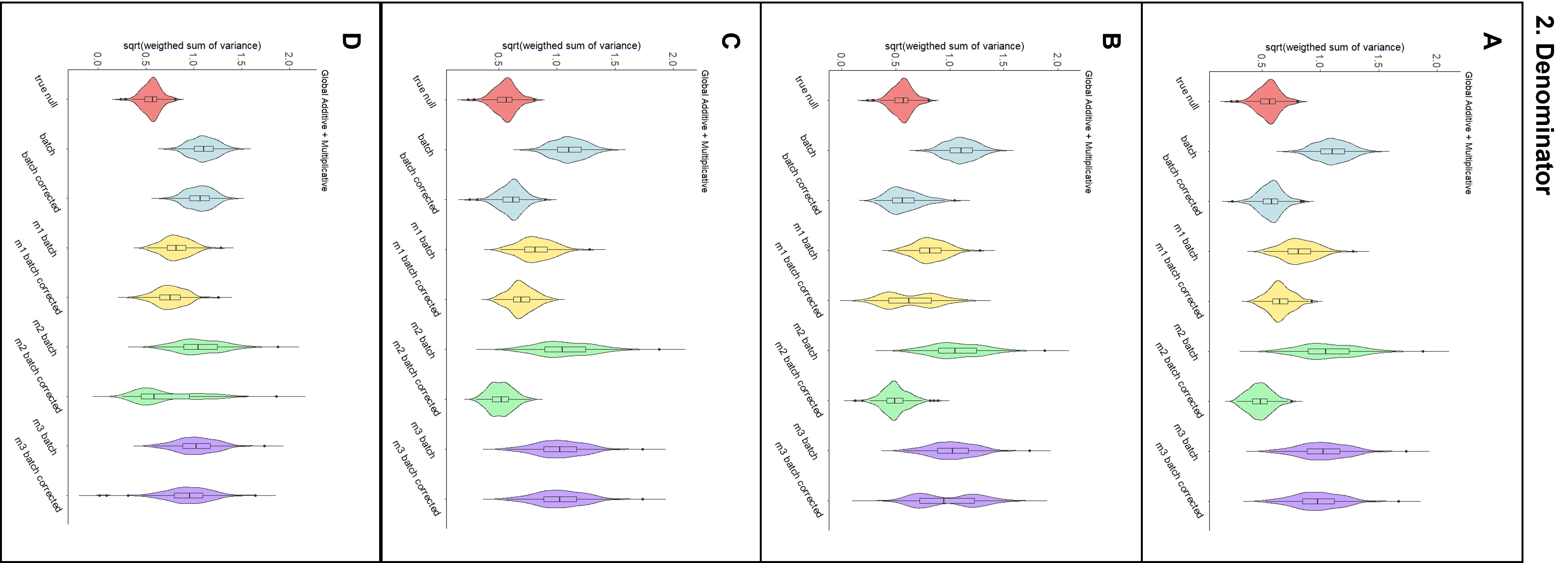
**

**Figure S8.** t-statistics split into **A.** numerator (delta means) and **B.** denominator (standard error of the mean) for Initial Simulation.


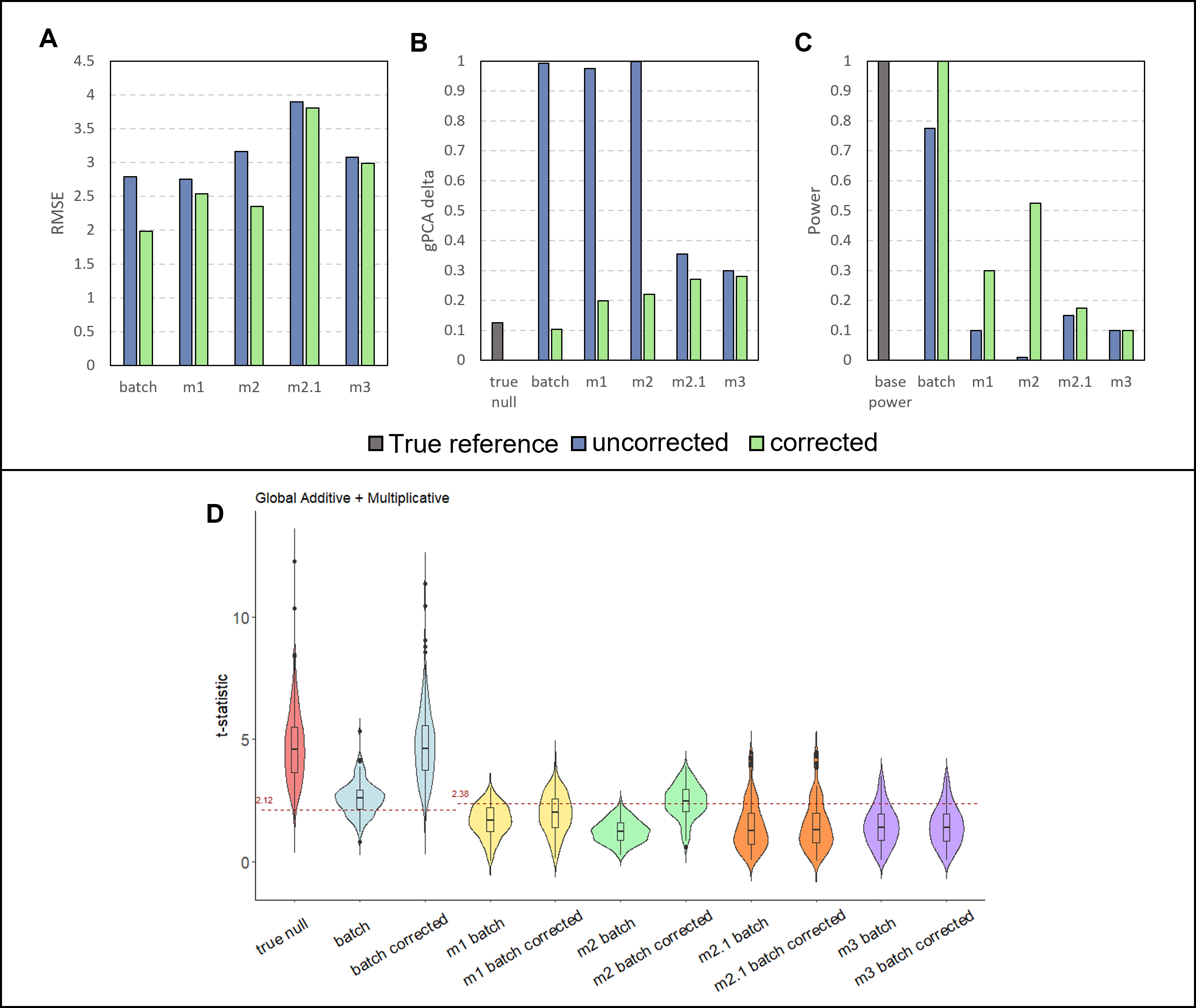


**Figure S9. A.** RMSE, **B.** gPCA delta, **C.** Power, **D.** t-statistics results for imputation strategies (M1 to M3), including M2.1 (imputation based on same class and batch), show that while M2.1 yield t-statistics more similar to original data, it did not outperform M2 in terms of RMSE, gPCA and power.


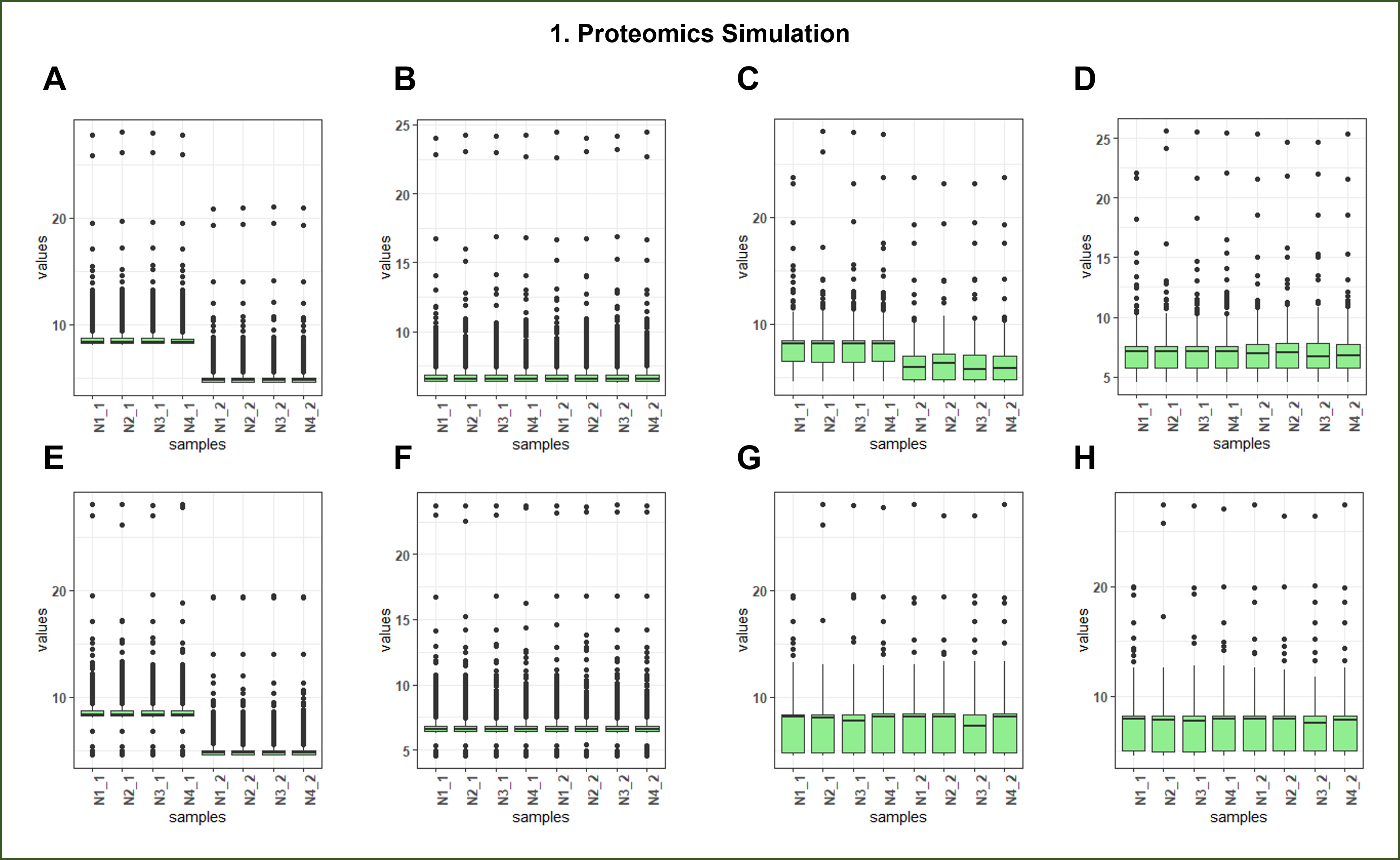

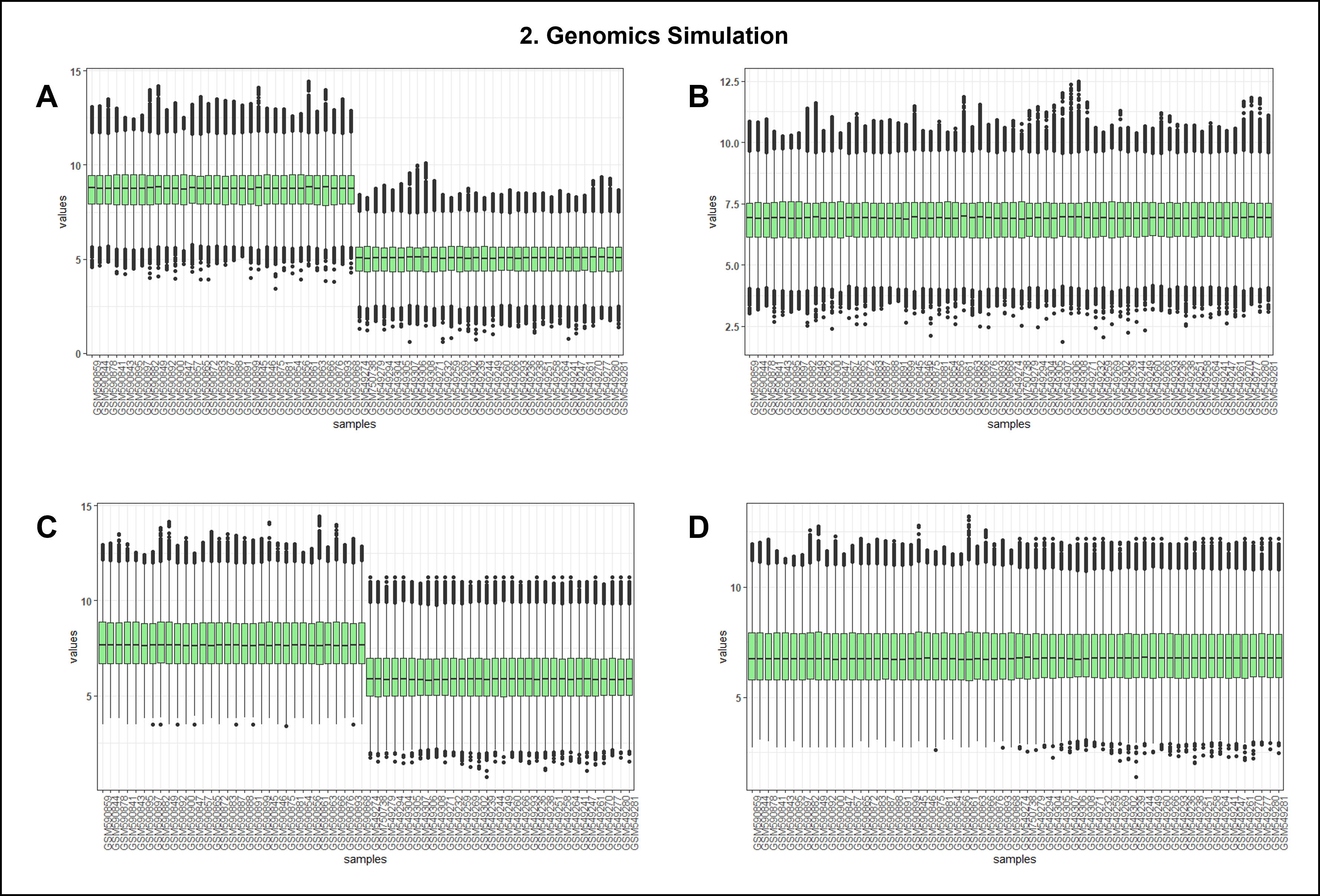

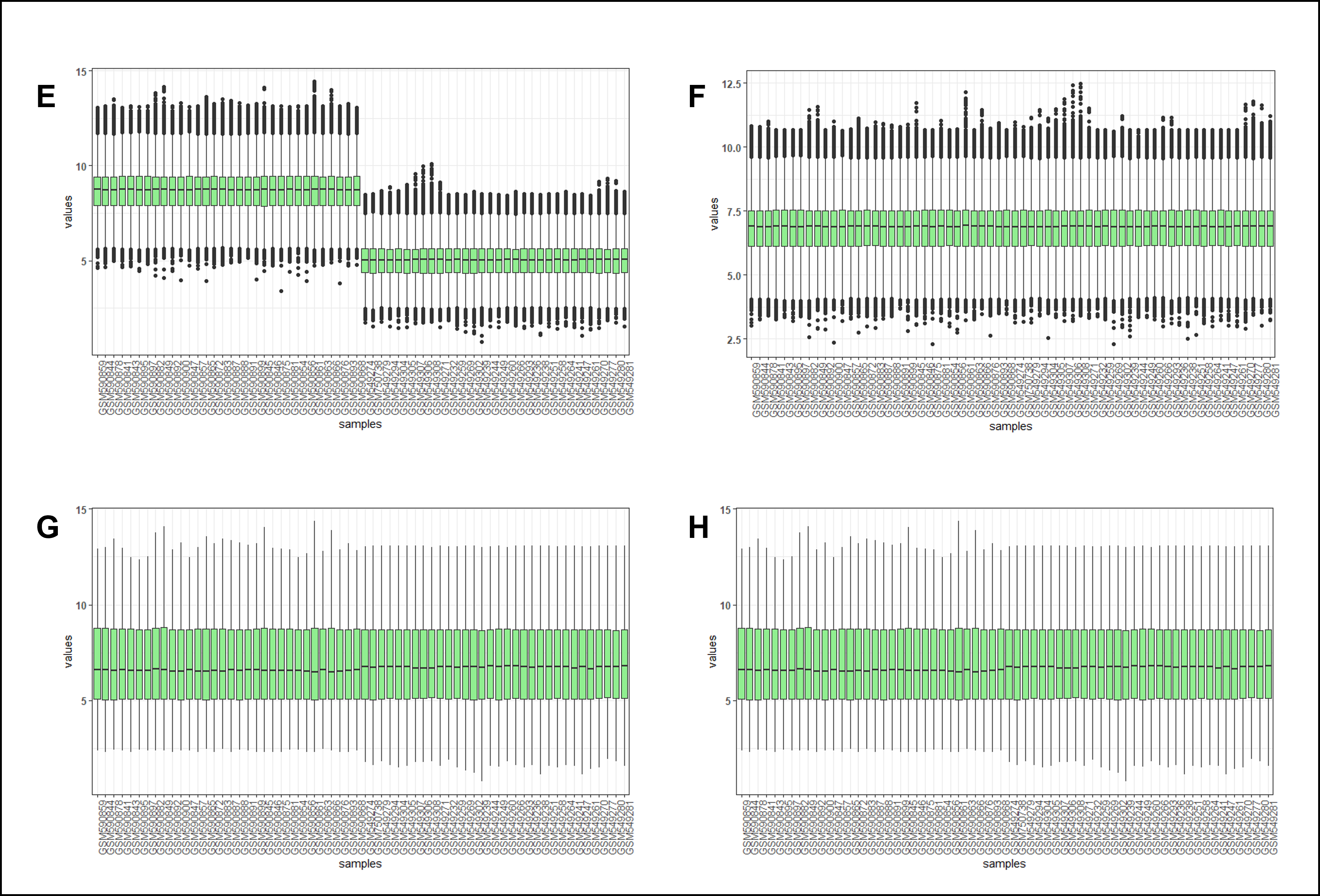


**Figure S10.** Sample Boxplots for **A.** Proteomics Simulation and **B.** Genomics Simulation although batch effects appear to be “mitigated” in the PCA scatterplots, M1 and M3 result in increased noise (i.e. larger interquartile range) in the data.

**
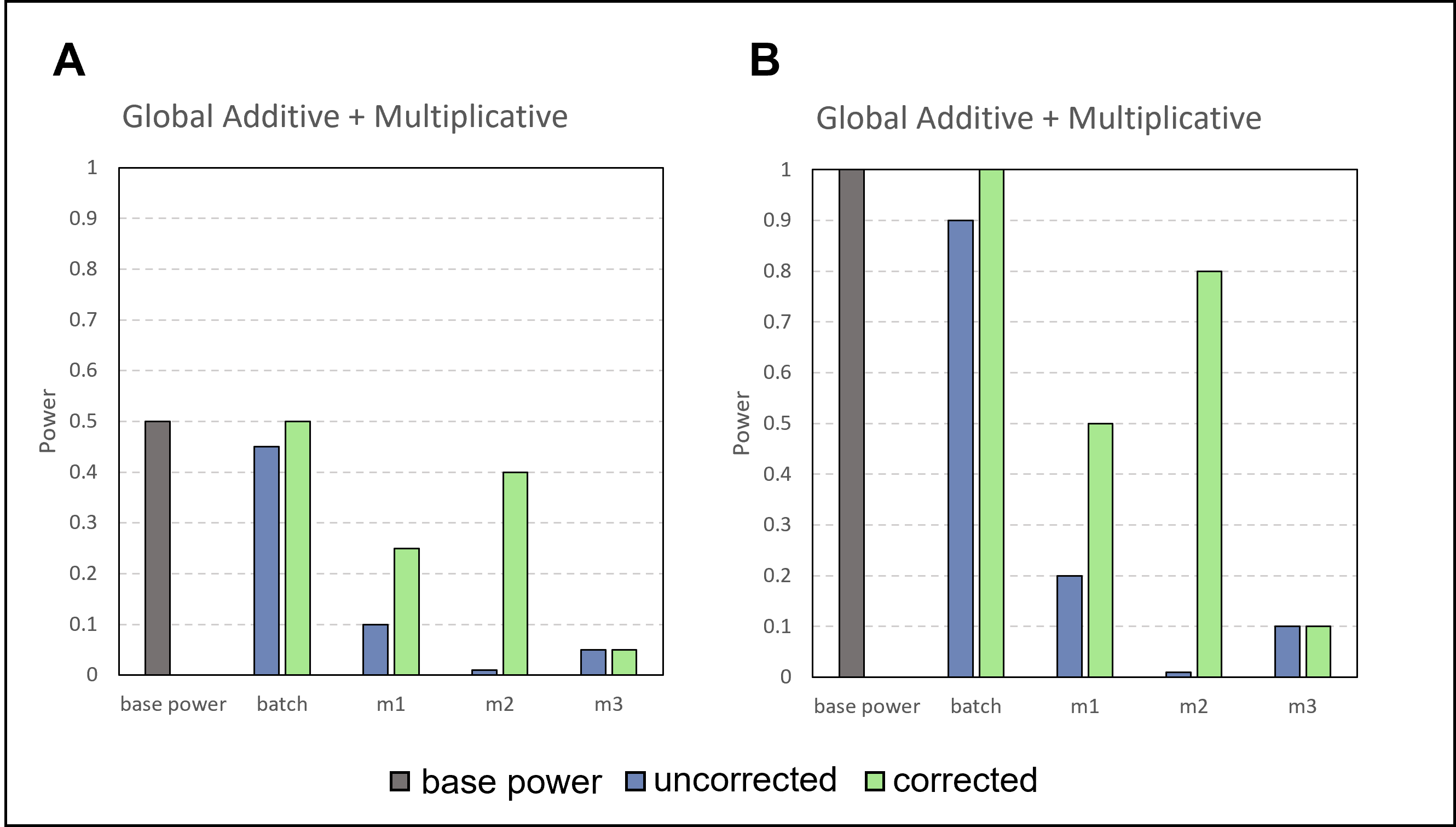
Figure S11. A.** Power and **B.** Recall for reduced genomics data (20x20 matrix) show that after reducing sample size of genomics data, M2 batch corrected no longer performs as well as batch corrected


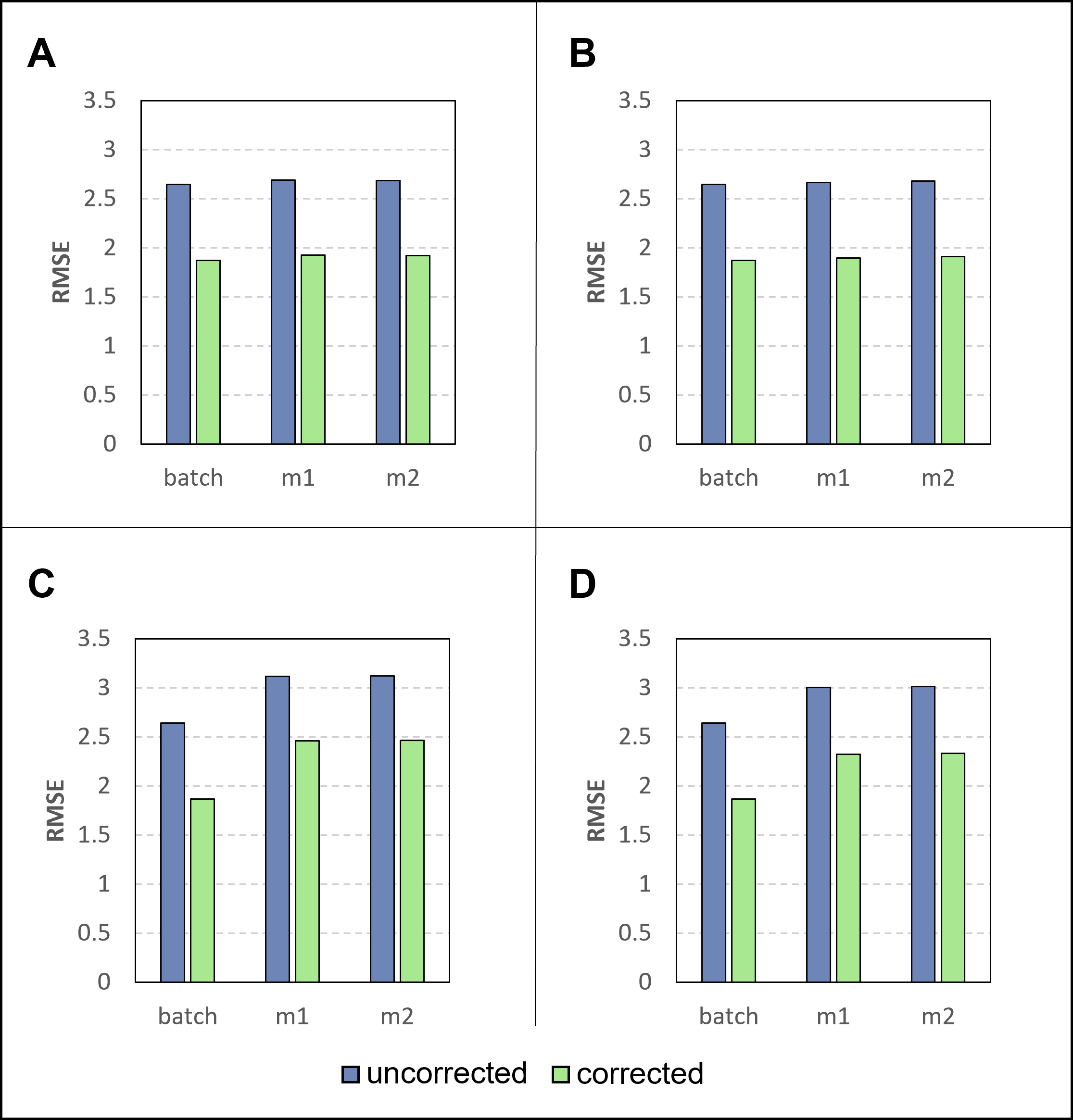


Figure S12. RMSE results of real datasets imputed by KNN and MICE. A. Proteomics Simulation with KNN imputation. B. Proteomics Simulation with MICE imputation. C. Genomic Simulation with KNN imputation. D. Genomic Simulation with MICE imputation.


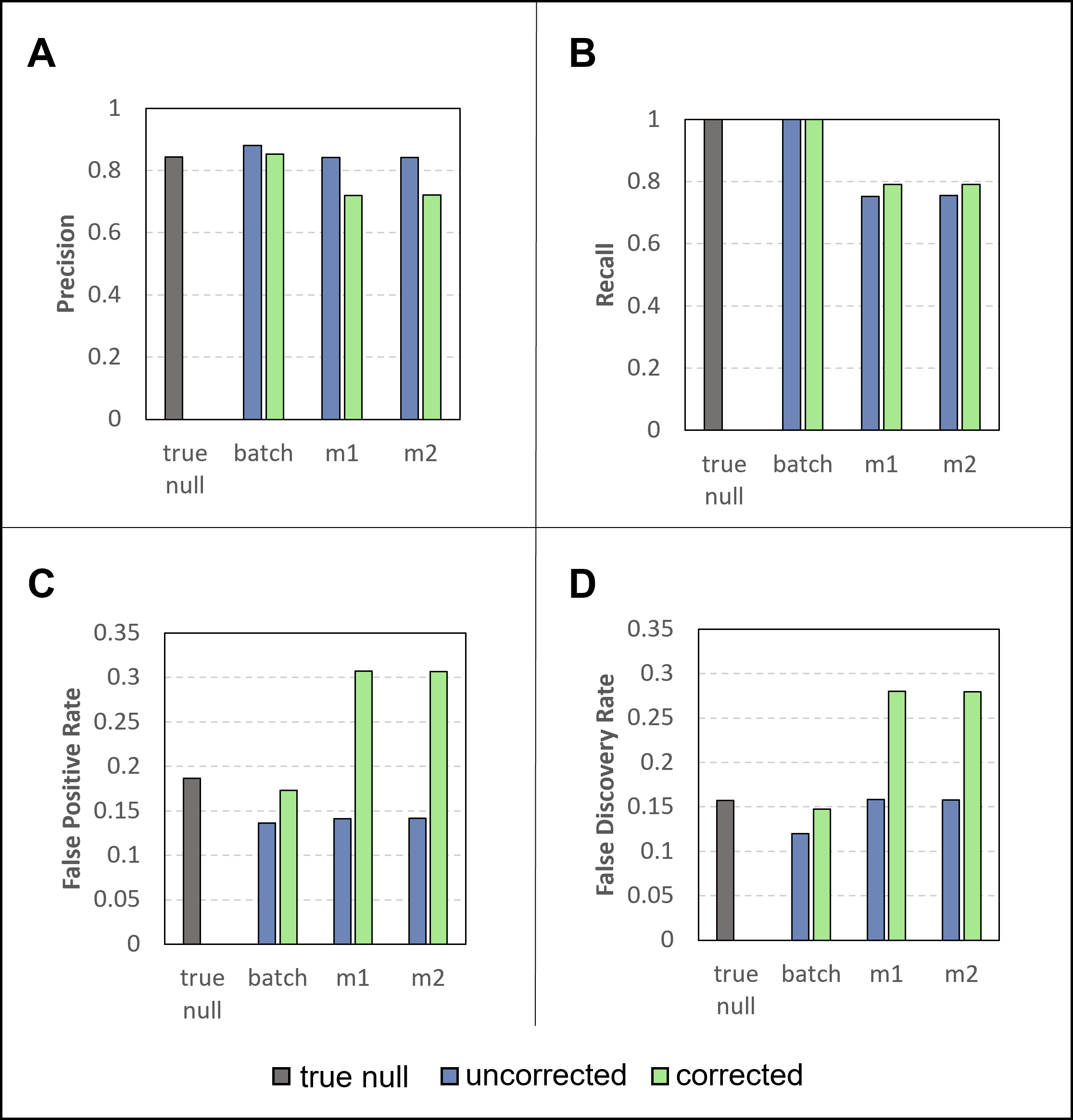


Figure S13. Performance Metrics for KNN imputed Genomics Simulation. Higher values indicate better performance: **A.** Precision **B.** Recall. Lower values indicate better performance: **C.** False Positive Rate (FPR) **D.** False Discovery Rate (FDR).


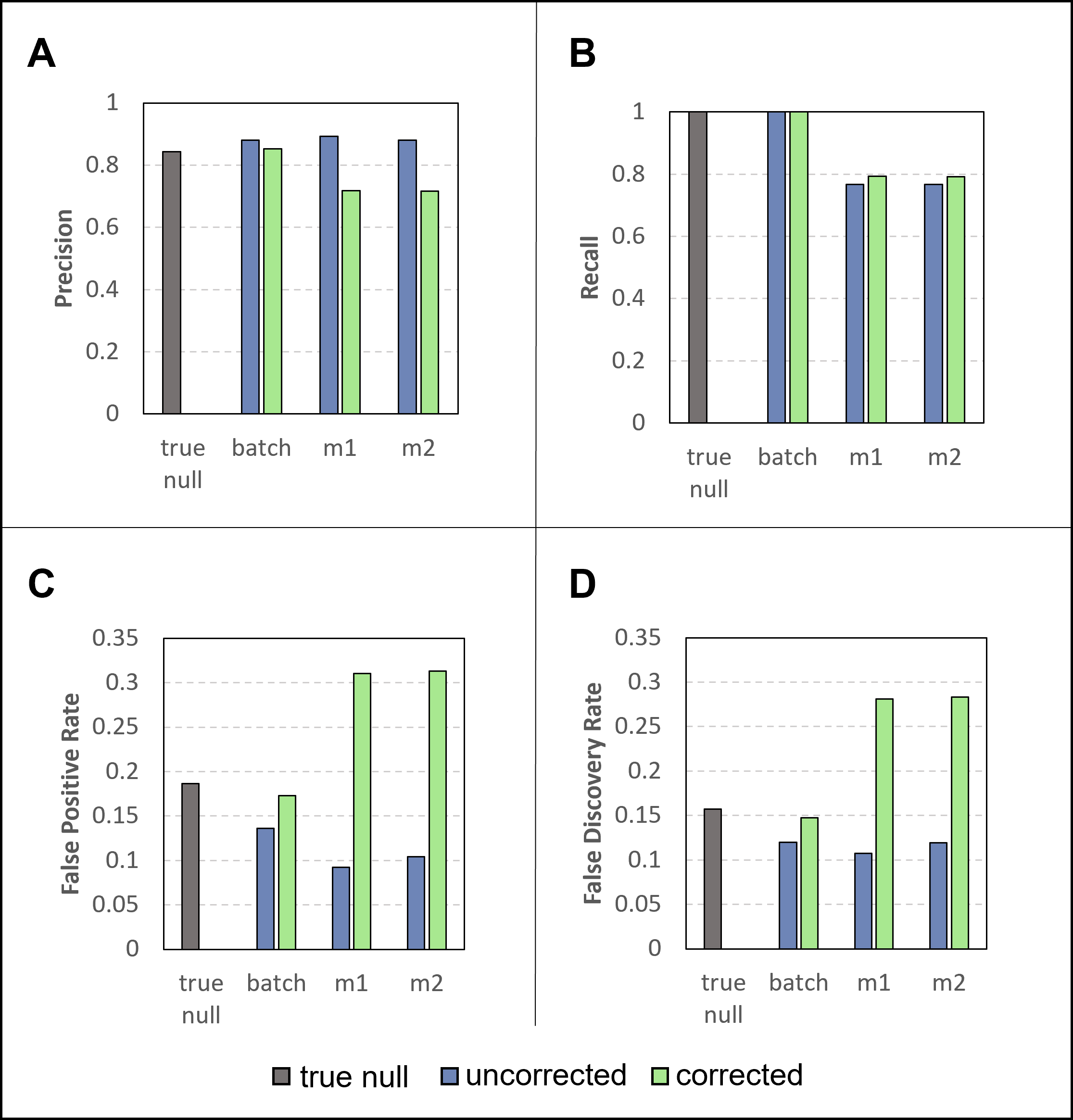


Figure S14. Performance Metrics for MICE imputed Genomics Simulation. Higher values indicate better performance: **A.** Precision **B.** Recall. Lower values indicate better performance: **C.** False Positive Rate (FPR) **D.** False Discovery Rate (FDR).
